# MGDrivE 2: A simulation framework for gene drive systems incorporating seasonality and epidemiological dynamics

**DOI:** 10.1371/journal.pcbi.1009030

**Published:** 2021-05-21

**Authors:** Sean L. Wu, Jared B. Bennett, Héctor M. Sánchez C., Andrew J. Dolgert, Tomás M. León, John M. Marshall

**Affiliations:** 1 Divisions of Epidemiology and Biostatistics, School of Public Health, University of California, Berkeley, California, United States of America; 2 Biophysics Graduate Group, Division of Biological Sciences, College of Letters and Science, University of California, Berkeley, California, United States of America; 3 Institute for Health Metrics and Evaluation, Seattle, Washington, United States of America; 4 Innovative Genomics Institute, University of California, Berkeley, California, United States of America; bioinformatics, GERMANY

## Abstract

Interest in gene drive technology has continued to grow as promising new drive systems have been developed in the lab and discussions are moving towards implementing field trials. The prospect of field trials requires models that incorporate a significant degree of ecological detail, including parameters that change over time in response to environmental data such as temperature and rainfall, leading to seasonal patterns in mosquito population density. Epidemiological outcomes are also of growing importance, as: i) the suitability of a gene drive construct for release will depend on its expected impact on disease transmission, and ii) initial field trials are expected to have a measured entomological outcome and a modeled epidemiological outcome. We present MGDrivE 2 (Mosquito Gene Drive Explorer 2): a significant development from the MGDrivE 1 simulation framework that investigates the population dynamics of a variety of gene drive architectures and their spread through spatially-explicit mosquito populations. Key strengths and fundamental improvements of the MGDrivE 2 framework are: i) the ability of parameters to vary with time and induce seasonal population dynamics, ii) an epidemiological module accommodating reciprocal pathogen transmission between humans and mosquitoes, and iii) an implementation framework based on stochastic Petri nets that enables efficient model formulation and flexible implementation. Example MGDrivE 2 simulations are presented to demonstrate the application of the framework to a CRISPR-based split gene drive system intended to drive a disease-refractory gene into a population in a confinable and reversible manner, incorporating time-varying temperature and rainfall data. The simulations also evaluate impact on human disease incidence and prevalence. Further documentation and use examples are provided in vignettes at the project’s CRAN repository. MGDrivE 2 is freely available as an open-source R package on CRAN (https://CRAN.R-project.org/package=MGDrivE2). We intend the package to provide a flexible tool capable of modeling gene drive constructs as they move closer to field application and to infer their expected impact on disease transmission.

## Author summary

Malaria, dengue and other mosquito-borne diseases continue to pose a major global health burden through much of the world. Currently available tools, such as insecticides and antimalarial drugs, are not expected to be sufficient to eliminate these diseases from highly-endemic areas, hence there is interest in novel strategies including genetics-based approaches. In recent years, the advent of CRISPR-based gene-editing has greatly expanded the range of genetic control tools available, and MGDrivE 1 (Mosquito Gene Drive Explorer 1) was proposed to simulate the dynamics of these systems through spatially-structured mosquito populations. As the technology has advanced and potential field trials are being discussed, models are now needed that incorporate additional details, such as life history parameters that respond to daily and seasonal environmental fluctuations, and transmission of pathogens between mosquito and vertebrate hosts. Here, we present MGDrivE 2, a gene drive simulation framework that significantly improves upon MGDrivE 1 by addressing these modeling needs. MGDrivE 2 has also been reformulated as a stochastic Petri net, enabling model specification to be decoupled from simulation, making it easier to adapt the model for application to other insect and mammalian species.

This is a *PLOS Computational Biology* Software paper.

## 1. Introduction

Interest in gene drive technology has continued to grow in recent years as a range of promising new constructs have been developed in the lab and discussions have moved towards implementing field trials in some cases. Recently developed systems include a CRISPR-based homing system intended for population suppression targeting the *doublesex* gene in *Anopheles gambiae*, the main African malaria vector [1], a split gene drive system intended for confineable and transient population replacement in *Aedes aegypti*, the main vector of dengue, chikungunya and Zika viruses [2], and CRISPR-based homing systems intended for population replacement in *An*. *gambiae* [3] and *Anopheles stephensi*, the main malaria vector in urban India [4].

As the technology advances and potential field trials are discussed [5], models are needed that incorporate additional ecological detail, including parameters that change over time in response to environmental variables such as temperature and rainfall, as well as models linking entomological and epidemiological outcomes [6]. Many insects, including mosquitoes, display a high degree of seasonality in their population dynamics, as development time from one life stage to another, and mortality rates associated with each life stage, vary with temperature and other environmental variables [7]. For *An*. *gambiae* and several other mosquito disease vectors, population size varies largely in response to recent rainfall, which creates pools of standing water and hence enhanced carrying capacity of the environment for mosquito larvae [8]. Seasonal changes in temperature and rainfall thus lead to seasonal changes in mosquito population density and consequent disease transmission, which must be accounted for in disease control strategies.

Models of disease transmission are also becoming increasingly relevant to models of gene drive dynamics, as: i) the readiness of a gene drive system for field trials will be determined in part by its expected (i.e. modeled) epidemiological impact, and ii) initial field trials are expected to have a measured entomological outcome alongside a modeled epidemiological outcome [5]. Given the potential for a non-localized gene drive system to spread broadly, it has been acknowledged that such constructs at the trial stage should be expected to cause a significant reduction in disease transmission, as even a confined trial could lead to wide-scale spread for an effective system [5]. Therefore, readiness for field trials should be determined by alignment with a target product profile (TPP) and/or list of preferred product characteristics (PPCs) that include expected impact on disease transmission [6]. Models that incorporate both gene drive and epidemiological dynamics can account for local malaria or arboviral transmission dynamics and specify gene drive construct parameters that achieve the desired level of epidemiological control.

Previously, we developed the MGDrivE 1 modeling framework to model the population dynamics of a variety of genetics-based and biological control systems and their spread through spatially-explicit populations of mosquitoes, or insects having a similar life history [9]. Here, we present MGDrivE 2, which significantly improves upon the capabilities of MGDrivE 1 by addressing the above-mentioned considerations, namely: i) the ability of parameter values to change over time, and hence to model seasonal population dynamics, and ii) the incorporation of an epidemiology module that can accommodate pathogen transmission between humans and mosquitoes. Minor additional improvements have been made to the inheritance, life history and landscape modules of the framework to reflect advances in these fields; for instance, a more resolved understanding of maternal deposition of Cas protein for CRISPR-based gene drive systems has been incorporated [10]. Models in MGDrivE 2 are represented as a stochastic Petri net (SPN), which has both computational and architectural benefits: model specification is separate from simulation, models can be efficiently stored and updated in memory, and a wealth of fast simulation algorithms from other fields can be used [11].

In this paper, we describe the key developments implemented in MGDrivE 2. We then demonstrate the application of the framework to the disease control impact of a CRISPR-based split gene drive system intended to drive a disease-refractory gene into a population in a confinable and reversible manner, and conclude with a discussion of future needs and applications for simulation packages in the field of gene drive modeling.

## 2. Design and implementation

**Fig 1:**
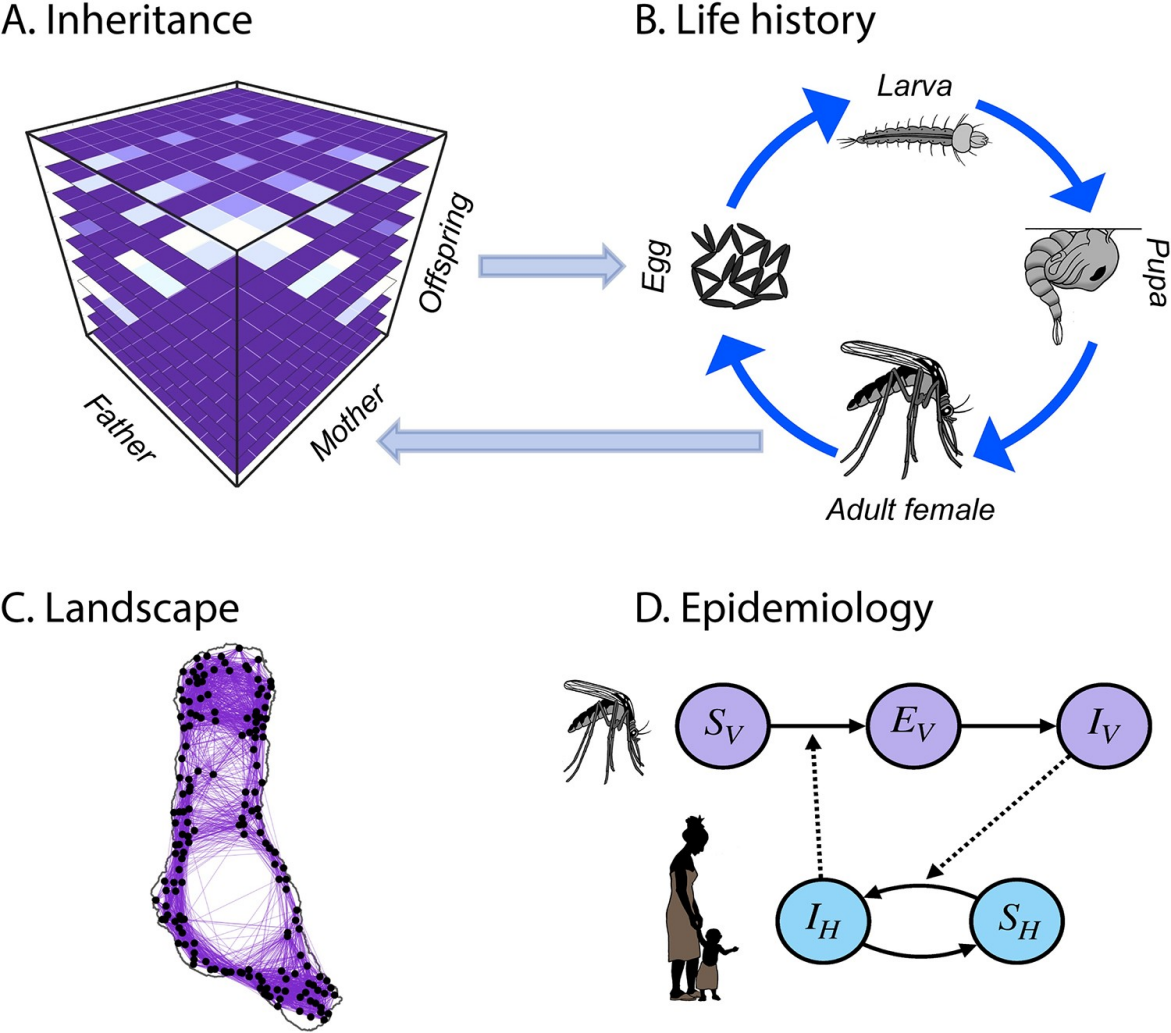
Modules in the MGDrivE 2 framework. **(A)** Genetic inheritance is embodied by a three-dimensional tensor referred to as an “inheritance cube.” Maternal and paternal genotypes are depicted on the *x* and *y*-axes and offspring genotypes on the *z*-axis. **(B)** Mosquito life history is modeled according to an egg-larva-pupa-adult (female and male) life cycle in which density dependence occurs at the larval stage, and life cycle parameters may vary as a function of environmental variables over time. Genotypes are tracked across all life stages, and females obtain a composite genotype upon mating—their own and that of the male they mate with. Egg genotypes are determined by the inheritance cube. **(C)** The landscape represents a metapopulation in which mosquitoes are distributed across population nodes and move between them according to a dispersal kernel. Population sizes and movement rates may vary as a function of environmental variables. **(D)** The epidemiology module describes reciprocal transmission of a vector-borne pathogen between mosquitoes and humans. This requires modeling human as well as mosquito populations, and the number of individuals having each infectious state. Epidemiological parameters may vary as a function of environmental variables.

MGDrivE 2 is a significant extension of and development from MGDrivE 1, a model for the spread of gene drive systems through spatially-explicit mosquito populations. The MGDrivE 2 model incorporates: i) an “inheritance module” that describes the distribution of offspring genotypes for given maternal and paternal genotypes, ii) a “life history module” that describes the development of mosquitoes from egg to larva to pupa to adult, iii) a “landscape module” that describes the distribution and movement of mosquitoes through a metapopulation, and iv) an “epidemiology module” that describes pathogen transmission between mosquitoes and humans (Fig 1). The framework is formulated as a SPN that can be mapped to a continuous-time Markov process in which model parameters may vary over time. It can also be implemented as a deterministic model via mean-field approximation of the stochastic model [12].

The core framework is developed in R (https://www.r-project.org/). The SPN framework enables separation of model components, allowing users to modify code on a component-by-component basis as needed for model specification or computational speed. We now describe the model extensions and developments from MGDrivE 1 to 2 in more detail. Full details of the MGDrivE 2 model framework are provided in the S1 Text.

### 2.1. Time-dependent parameters and seasonality

The incorporation of time-dependent parameters represents a significant improvement of the MGDrivE 2 modeling framework. In MGDrivE 1, the mosquito life history module follows the lumped age-class model of Hancock and Godfray as adapted by Deredec *et al*. [13], which describes development from egg to larva to pupa to adult using delay-difference equations. The delay framework allows development times to be modeled as fixed rather than exponentially-distributed; however, it is not compatible with time-varying parameters as these could vary during the delay. In MGDrivE 2, the discrete-time, fixed-delay framework of MGDrivE 1 is replaced by a continuous-time implementation in which each life stage is divided into a series of substages. For a single substage, the development time is exponentially-distributed; but as the number of substages increases, the distribution of development times becomes concentrated around the mean. Specifically, if a life stage with a mean development time of 1/*d* is divided into a series of *n* substages, the new development times are Erlang-distributed with mean, *1/d*, and variance, 1/(*dn*^2^), or equivalently, with shape parameter, *n*, and rate parameter, *d*/*n*. The development time, *d*(*t*), may also vary over time, *t*; however the number of substages, *n*, and hence the mean-variance relationship for development times, must remain constant within a simulation.

Most importantly, the new model implementation allows any model parameter to vary with time, enabling the framework to account for seasonal variation in development times and mortality rates due to environmental dependencies. Temperature is known to strongly influence development times for juvenile mosquito stages, and mortality rates for all mosquito life stages [7,14], and rainfall is known to influence the carrying capacity of the environment for larvae, and therefore density-dependent larval mortality rates [8,15]. The new model formulation allows these parameters to vary in continuous time in response to environmental data, and hence for seasonal variations in temperature and rainfall to drive seasonal variations in mosquito population density.

Parameters defining other modules of the model—inheritance, landscape and epidemiology—are also able to vary over time within the new model formulation. For instance, gene drive systems under the control of temperature-dependent promoters [16,17] may have time-varying homing efficiencies, mosquito movement rates may vary seasonally in response to temperature and other environmental factors [18], and epidemiological parameters such as the extrinsic incubation period (EIP) and pathogen transmission probabilities from human-to-mosquito and mosquito-to-human are all known to display seasonal variation through temperature dependence [7,14].

### 2.2. Epidemiology module

**Fig 2:**
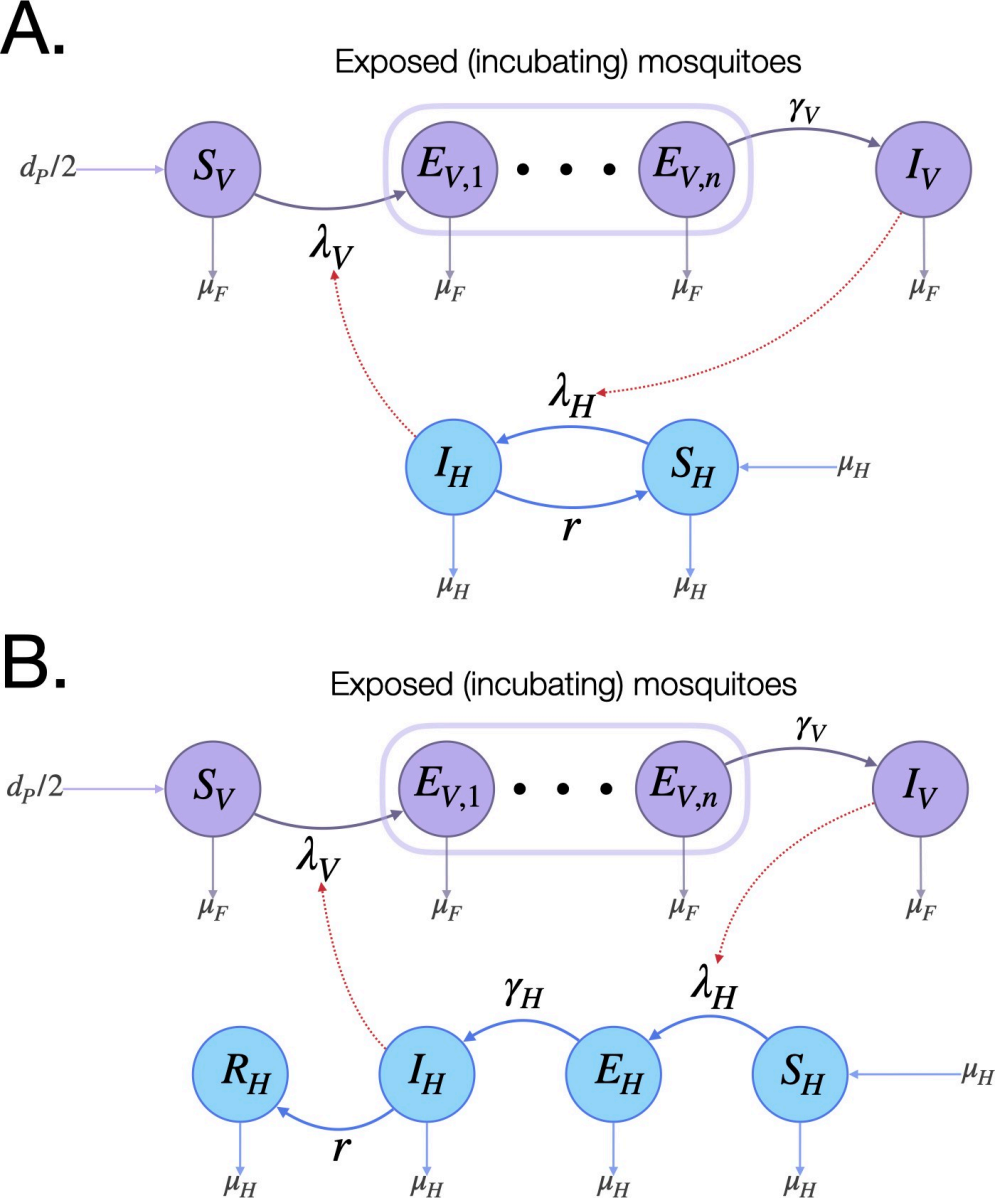
Epidemiology module. MGDrivE 2 includes two basic models for reciprocal pathogen transmission between mosquitoes and humans—one for malaria **(A)**, and one for arboviruses **(B)**. In both cases, female mosquitoes emerge from pupae at a rate equal to *d*_*P*_/2 as susceptible adults (S_V_), become exposed/latently infected (E_V,1_) at a rate equal to the force of infection in mosquitoes, *λ*_*V*_, and progress to infectiousness (I_V_) through the extrinsic incubation period (EIP = 1/*γ*_*V*_), which is divided into *n* bins to give an Erlang-distributed dwell time. The mortality rate, *μ*_*F*_, is the same for female mosquitoes in each of these states. For malaria **(A)**, susceptible humans (S_H_) become infected/infectious (I_H_) at a rate equal to the force of infection in humans, *λ*_*H*_, and recover at rate *r*, becoming susceptible again. For arboviruses **(B)**, susceptible humans (S_H_) become exposed/latently infected (E_H_) at a rate equal to *λ*_*H*_, progress to infectiousness (I_H_) at rate equal to *γ*_*H*_, and recover (R_H_) at rate, *r*. Infection dynamics couple the mosquito and human systems via the force of infection terms; *λ*_*V*_ is a function of I_H_, and *λ*_*H*_ is a function of I_V_, shown via red edges.

The epidemiology module describes reciprocal transmission of a vector-borne pathogen between mosquitoes and humans. This requires modeling of both vector and human populations, as well as an attribute describing the number of individuals in the vector and human populations having each infectious state (Fig 2). To model malaria, the Ross-Macdonald model is included, which has susceptible (S_V_), exposed/latently infected (E_V_), and infectious (I_V_) states for mosquitoes, and susceptible (S_H_), and infected/infectious (I_H_) states for humans [19,20]. Malaria infection in humans is described by an SIS model, in which humans become infected at a per-capita rate equal to the “force of infection” in humans, *λ*_*H*_, and recover at a rate, *r*. Malaria infection in mosquitoes is described by an SEI model, in which adult mosquitoes emerge from pupae in the susceptible state, become exposed and latently infected at a per-capita rate equal to the force of infection in mosquitoes, *λ*_*V*_, and progress to infectiousness at a rate equal to *γ*_*V*_. The force of infection in humans, *λ*_*H*_, is proportional to the number of mosquitoes that are infectious, *I*_*V*_ and the force of infection in mosquitoes, *λ*_*V*_, is proportional to the fraction of humans that are infectious, *I*_*H*_/*N*_*H*_, where *N*_*H*_ is the human population size. Since an exponentially-distributed EIP leads to some mosquitoes having unrealistically brief incubation periods, we divide the E_V_ state into a series of *n* sub-states, as described in section 2.1, leading to the EIP being Erlang-distributed with shape parameter, *n*, and rate parameter, *γ*_*V*_/*n* [21]. Finally, transmission parameters may be tied to specific mosquito genotypes—for instance, an antimalarial effector gene may be associated with a human-to-mosquito or mosquito-to-human transmission probability of zero.

To model arboviruses such as chikungunya, Zika and single serotypes of dengue virus, we include an SEIR model for human transmission, in which the human states are: susceptible (S_H_), exposed/latently infected (E_H_), infectious (I_H_), and removed/recovered (R_H_) [22,23]. The E_H_ and R_H_ states are included because arboviruses are generally thought to be immunizing, and have latent periods that tend to be on a similar timescale to the duration of infectiousness. Humans become latently infected at a per-capita rate equal to *λ*_*H*_, progress to infectiousness at rate, *γ*_*H*_, and recover at rate, *r*. For mosquito transmission, the SEI model with an Erlang-distributed EIP is used again. Further details of the mathematical formulation of both the malaria and arbovirus models are provided in the S1 Text. The extensibility of the SPN framework means that more complex epidemiological models can be developed and implemented by users.

Modeling vector-borne disease transmission within a metapopulation framework generally requires each population node in the network to have both a defined mosquito and human population size. Since the mosquito vectors we are interested in are anthropophilic, they tend to coexist with humans, so human population sizes and state distributions can be attributed to the same nodes at which mosquito populations are defined; however, MGDrivE 2 also includes the possibility of human-only and mosquito-only nodes. Mosquito-only nodes could represent sites with only non-human vertebrates from which mosquitoes bloodfeed, while human-only nodes could represent locations unsuitable for mosquitoes. As mosquitoes are able to move between nodes in the metapopulation, so can humans. This is an important factor to include, as human movement has been shown to drive the spatial transmission of mosquito-borne diseases such as dengue virus [24].

### 2.3. Other extensions to inheritance, life history and landscape modules

Additional functionality has been included in the inheritance and life history modules of the MGDrivE framework since publication of version 1.0. The inheritance module is unchanged, and inheritance “cubes,” describing the distribution of offspring genotypes given maternal and paternal genotypes for a given genetic element, are usable in both versions. Several new inheritance cubes have been made available, including: a) homing-based remediation systems, including ERACR (Element for Reversing the Autocatalytic Chain Reaction) and e-CHACR (Erasing Construct Hitchhiking on the Autocatalytic Chain Reaction) [25,26], and b) newly proposed drive systems capable of regional population replacement, including CleaveR (Cleave and Rescue) [27] and TARE (Toxin-Antidote Recessive Embryo) drive [28].

In the life history module, we have provided two alternative parameterizations of a quadratic density-dependent larval mortality rate function corresponding to logistic and Lotka-Volterra ecological models. For mosquito vectors such as *Ae*. *aegypti* and *An*. *gambiae*, density-dependence is thought to act at the larval stage due to increased resource competition at higher larval densities [8,15]. The adult population size, *N*, is used to determine the value of *K*, the larval density at which the larval mortality rate is twice the density-independent mortality rate at a given patch, which produces the appropriate equilibrium population size. For the logistic model, the per-capita larval mortality rate is given by *μ*_*L*_ (1 + *L*(*t*)/*K*), where *μ*_*L*_ is the density-independent larval mortality rate, and *L*(*t*) is the total larval population size for the patch at time *t*. For the Lotka-Volterra model, the per-capita larval mortality rate is given by *μ*_*L*_ + *α L*(*t*), where *α* is the density-dependent term. While related by the expression, *α = μ*_*L*_*/K*, these two models provide an example of how different functional forms can be used for rates in MGDrivE 2, and may serve as a template for incorporating more elaborate density-dependent functions.

In the landscape module, movement through the network of population nodes is again determined by a dispersal kernel; however, due to the continuous-time nature of MGDrivE 2, movement between patches is described by a rate rather than a probability. MGDrivE 2 provides functions to map transition probability matrices from MGDrivE 1, such as the zero-inflated exponential or lognormal dispersal kernels, to continuous-time transition rate matrices for MGDrivE 2. These mapping functions may also be applied to transition probability matrices derived from empirical or simulated data. The mathematical mapping between the rate matrix of MGDrivE 2 and the transition probability matrix of MGDrivE 1 is provided in the S1 Text.

### 2.4. Stochastic Petri net formulation

The most fundamental change from MGDrivE 1 to 2 is restructuring the model as a SPN [29]. Adopting a SPN framework has several benefits. First, SPNs allow the mathematical specification of a model to be decoupled from its algorithmic implementation, allowing users to leverage extensive sampling algorithms from the physical and chemical simulation communities for efficient computation [11,30]. Second, SPNs have a well-established and consistent formalism, allowing them to be readily understood and modified by anyone familiar with this [31]. And third, SPNs are isomorphic to continuous-time Markov chains (CTMCs), meaning that model parameters can be time-varying, including Erlang-distributed aquatic stage durations and the pathogen’s EIP.

**Fig 3:**
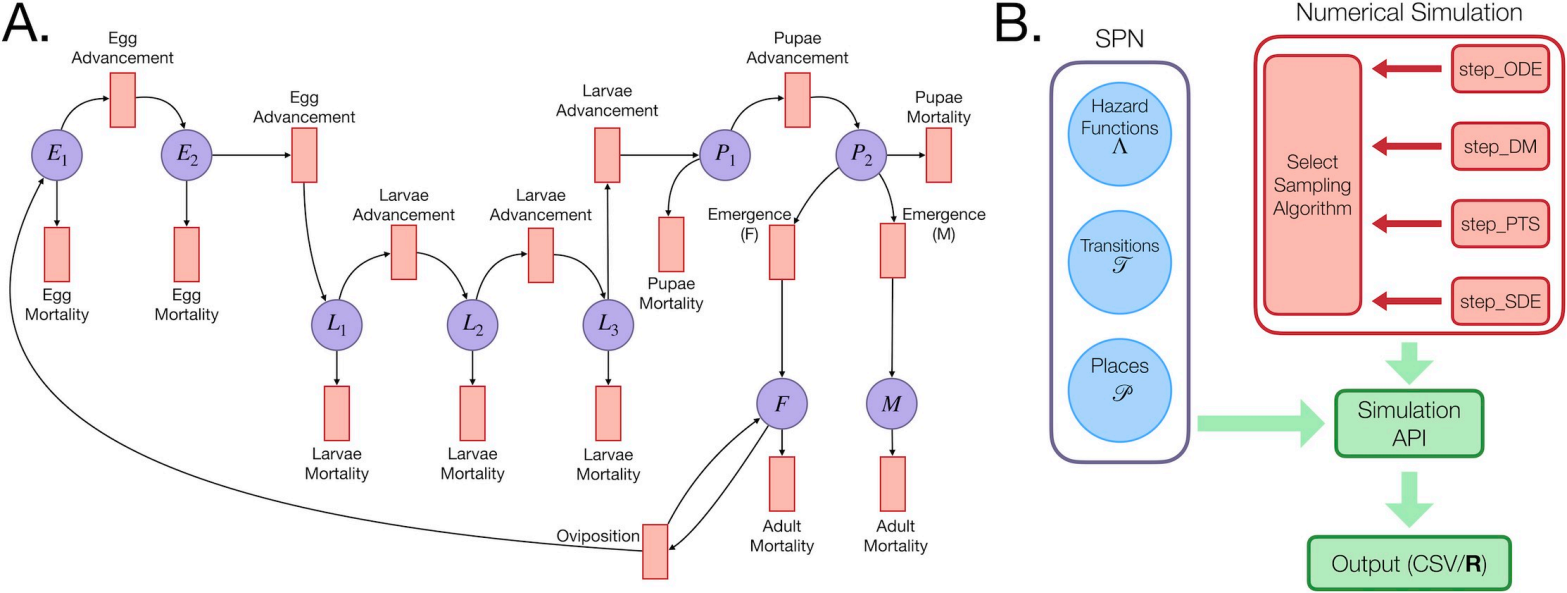
Stochastic Petri net (SPN) implementation of MGDrivE 2. **(A)** Petri net representation of the life history module. The set of purple circles corresponds to places, *P*, and red rectangles to transitions, *T*. This Petri net shows a model in which development times for the egg stage are Erlang-distributed with shape parameter *n* = 2, and for the larval stage are Erlang-distributed with shape parameter *n* = 3. Population dynamics are derived directly from this graph. E.g. The transition corresponding to oviposition has one edge beginning at F, meaning at least one female mosquito must be present for oviposition to occur. When oviposition occurs, a token is added to E_1_ (new eggs are laid) and a token is returned to F. **(B)** Conceptual representation of the SPN software architecture showing the separation between the model representation (blue circles) and set of sampling algorithms (red rectangles). These two components of the codebase meet at the simulation API, enabling users to match models and simulation algorithms interchangeably. Output may be returned as an array in R for exploratory work, or written to CSV files for large simulations.

A Petri net is a bipartite graph consisting of a set of places, *P*, and a set of transitions, *T*. Directed edges or “arcs” lead from places to transitions (input arcs) and from transitions to places (output arcs). The set of arcs that connect places to transitions and transitions to places can be denoted by two matrices whose entries are non-negative integers describing the weight of each arc. The places define the allowable state space of the model; however, in order to describe any particular state of the model, the Petri net must be given a marking, *M*, which is defined by associating each place with a non-negative integer number of tokens. In the language of CTMCs, *M* is referred to as a “state.” When a transition occurs, it induces a state change by “consuming” tokens in *M* given by the set of input arcs, and “producing” tokens in *M* according to the set of output arcs [32]. Each transition has a “clock process,” parameterized by a “hazard function” which defines that event’s current rate of occurrence. In MGDrivE 2, tokens represent an integer number of mosquitoes or humans, and the distribution of tokens (mosquitoes or humans) across states at time *t* defines a marking, *M*(*t*). A graphical representation of a Petri net for the mosquito life history module of MGDrivE 2 is depicted in Fig 3A, with a full description of the mathematical formalism provided in the S1 Text.

The code that generates the Petri net is independent of the code that simulates trajectories from it. Once the Petri net is stored as a set of sparse matrices, it is passed to a simulation application program interface (API) which allows trajectories to be simulated as ordinary differential equations (ODEs), stochastic differential equations (SDEs), or CTMCs (Fig 3B). Each of these are referred to as “step” functions, but are not limited to discrete time steps; these functions are responsible for updating the model between time points where the user requests output to be recorded. The ODE step function provides a deterministic approximation and interfaces with the numerical routines provided in the “deSolve” R package [33]. Three stochastic numerical routines are provided that treat the model as a continuous-time Markov process and provide different levels of approximation. The most straightforward method to sample trajectories is Gillespie’s direct method, which samples each event individually [34]. While statistically exact, this is prohibitively slow for medium-to-large population sizes. Two approximate stochastic methods are provided that have been widely used in the chemical physics literature: i) a second order continuous SDE approximation known as the chemical Langevin equation [35], and ii) a fixed-step tau-leaping method [36]. Both methods achieve substantial gains in computational speed at the expense of statistical accuracy. While the SDE approximation is often faster, tau-leaping retains the discrete character of the process it approximates and is usually the preferred technique. A full description of each of the numerical routines is provided in the S1 Text. In addition, we demonstrate how a user can write a custom simulation algorithm and incorporate it within the MGDrivE 2 codebase in the “Advanced Topics” vignette available at https://marshalllab.github.io/MGDrivE/docs_v2/articles/advanced_topics.html.

## 3. Results

To demonstrate how the MGDrivE 2 framework can be used to initialize and run a simulation of the spread of a gene drive system through a metapopulation with time-varying model parameters, including its implications for vector-borne pathogen transmission, we have provided vignettes with the package, available via installation from CRAN at https://CRAN.R-project.org/package=MGDrivE2 and additional examples and information on GitHub at https://marshalllab.github.io/MGDrivE/docs_v2/index.html. The vignettes provide extensive examples of how to use the software, including advanced features such as implementing custom time-varying rates and numerical simulation algorithms. They consist of a set of five “core” manuals that describe how to simulate population genetics and dynamics for a mosquito-only population and metapopulation, then how to incorporate SEI-SIS Ross-Macdonald malaria transmission dynamics in a population with humans included, and finally how to incorporate SEI-SEIR arbovirus transmission dynamics. Following these are three “advanced” manuals that introduce: i) how to process and analyze output from simulations that write to CSV files, ii) how users can write custom time-varying hazard functions, and iii) how a user might implement their own numerical simulation routine, using an explicit Euler method for ODEs as an example.

Here, we describe the application of the package to model the release of a split gene drive system designed to drive a malaria-refractory gene into an *An*. *gambiae* mosquito population with seasonal population dynamics and transmission intensity calibrated to a setting resembling the island of Grand Comore, Union of the Comoros. The split gene drive system resembles one engineered in *Ae*. *aegypti* [2], which was chosen as the only published split drive system in a mosquito vector to date. Split drive designs are well-suited to initial field trials of gene drive systems as they display transient drive activity before being eliminated by virtue of a fitness cost. The spatial spread of these systems is limited by the distance the host organism disperses while the drive system persists. In the split drive system explored here, two components—the Cas9 and guide RNA (gRNA)—are present at separate, unlinked loci, and a disease-refractory gene is linked to the gRNA. We assume that only one copy of the disease-refractory allele is required for it to block pathogen transmission. Four alleles are considered at the gRNA locus: an intact gRNA/refractory allele (denoted by “H”), a wild-type allele (denoted by “W”), a functional, cost-free resistant allele (denoted by “R”), and a non-functional or otherwise costly resistant allele (denoted by “B”). At the Cas9 locus, two alleles are considered: an intact Cas9 allele (denoted by “C”), and a wild-type allele (denoted by “W”). Full details of the inheritance dynamics are provided in Li *et al*. [2] and model parameters are summarized in S1 Table.

The life history module is parameterized with typical bionomic parameter values for *An*. *gambiae* (S1 Table), including mean-variance relationships describing the development times of juvenile life stages [37]. The carrying capacity of the environment for larvae is a function of recent rainfall, and the adult mortality rate is a function of temperature. Remotely sensed rainfall data for Grand Comore was obtained from the ERA5 dataset (https://www.ecmwf.int/en/forecasts/datasets/reanalysis-datasets/era5), and a mathematical relationship adapted from White *et al*. [8] was used to translate this to larval carrying capacity, assuming that half of the island’s carrying capacity was provided by permanent breeding sites (e.g. large cisterns) and half was provided by recent rainfall. Temperature data for Grand Comore was also obtained from the ERA5 dataset, and adult mortality was derived using methods described by Mordecai *et al*. [7]. Both climatological time series covered the six-year period beginning January 1, 2010. For the purpose of this demonstration, Grand Comore was treated as a single randomly mixing population, although simulations involving a more detailed landscape module are included in the vignettes.

The epidemiology module is parameterized with typical parameter values for *Plasmodium falciparum* transmission (S1 Table), human population size and life expectancy parameters from the National Institute of Statistics and Demographic Studies, Comoros [38], and is calibrated to local malaria prevalence estimates from the Malaria Atlas Project [39]. This calibration was achieved by multiplying the carrying capacity time series by a constant such that the average adult female mosquito population over a year sustained malaria transmission in the human population at the estimated local prevalence. Finally, we caution that these simulations are merely intended to demonstrate the software’s capabilities and that, while the simulations are parameterized with data from Grand Comore, they are not intended to provide an accurate forecast of local gene drive mosquito dynamics, or to imply approval of the intervention by the local population and regulatory agencies.

### 3.1. Simulation workflow

The code for this simulation is available at https://github.com/MarshallLab/MGDrivE/tree/master/Examples/SoftwarePaper2. We begin by loading the MGDrivE 2 package in R, as well as the package for the original MGDrivE simulation, which provides the inheritance cubes required for simulation of genetically-stratified mosquito populations. Next, we define model parameters, including the bionomic parameters of *An*. *gambiae* s.l., and demographic and epidemiological parameters specific to Grande Comore. To parameterize time-varying adult mosquito mortality (hourly) and larval carrying capacity (daily), we load CSV files containing those data as time series for the ten-year simulation period. We then use the base “stepFun()” function in R to create an interpolating function of those time-series data that will return a value for any time within the simulation period, which is required for calculation of hazard functions. More sophisticated interpolating functions, such as splines, may also be used. We also specify the inheritance cube at this point, as the number of modeled genotypes and distribution of offspring genotypes for given parental genotypes will be used to build the Petri net.

Next, we use functions from MGDrivE 2 to create the “places” and “transitions” of the Petri net, which are stored as lists in R and then converted into a sparse matrix representation used in the simulation code. Epidemiological dynamics and states are coded automatically by calling the functions that create the Petri net. In this case, “spn_P_epiSIS_node()” and “spn_T_epiSIS_node()” will generate the places and transitions for a single node model with SEI-SIS mosquito and human malaria transmission dynamics. Each transition has a tag that specifies the hazard function it requires. Following that, we write custom time-varying hazard functions for adult mosquito mortality and larval mortality (a function of carrying capacity). We provide a guided walkthrough of how a new user might write their own time-varying hazard function in the vignette “Simulation of Time-inhomogeneous Stochastic Processes.” Once the vector of hazard functions has been stored (as a list), we create the data frame that stores the times, genotypes, sex, and size of each release event.

With the construction of all model components necessary for the simulation, we call the simulation API which handles the details of simulating trajectories from the model. In this case, we chose the tau-leaping algorithm to sample stochastic trajectories, and to record output on a daily basis. MGDrivE 2 allows users to choose how model output is reported back—for exploratory or smaller simulations, users may return output directly to R as an array; however for larger simulations, it is often preferable to write directly to CSV files due to memory considerations, and MGDrivE 2 has sophisticated functions to both specify CSV output and process completed simulations.

### 3.2. Entomological population dynamics

**Fig 4:**
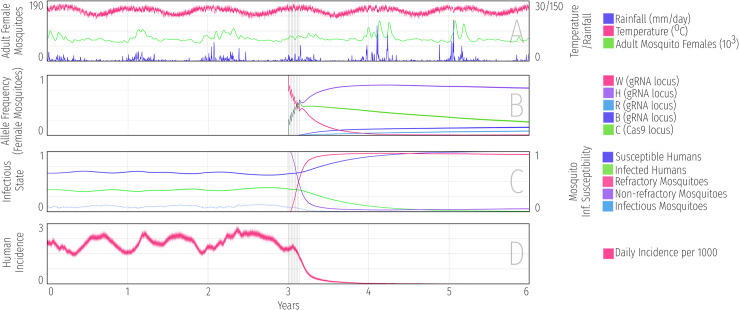
Example MGDrivE 2 simulations for a split gene drive system designed to drive a malaria-refractory gene in a confinable and reversible manner into an *An*. *gambiae* s.l. mosquito population with seasonal population dynamics and transmission intensity calibrated to a setting resembling the island of Grand Comore, Union of the Comoros. The split drive system resembles one recently engineered in *Ae*. *aegypti* [2]–the only split drive system in a mosquito vector to date. In the modeled system, two components–the Cas9 and guide RNA (gRNA)–are present at separate, unlinked loci, and a disease-refractory gene is linked to the gRNA. Four alleles are considered at the gRNA locus: an intact gRNA/refractory allele (denoted by “H”), a wild-type allele (denoted by “W”), a functional, cost-free resistant allele (denoted by “R”), and a non-functional or otherwise costly resistant allele (denoted by “B”). At the Cas9 locus, two alleles are considered: an intact Cas9 allele (denoted by “C”), and a wild-type allele (denoted by “W”). Model parameters describing the construct, mosquito bionomics and malaria transmission are summarized in S1 Table. **(A)** Climatological time-series data—temperature in red and rainfall in blue—that were used to calculate time-varying adult mosquito mortality rate and larval carrying capacity, respectively. The resulting adult female population size is shown in green. **(B)** Allele frequencies for adult female mosquitoes over the simulation period. Grey vertical bars beginning at year three denote eight consecutive weekly releases of 50,000 male mosquitoes homozygous for both the gRNA and Cas9 alleles (H and C, respectively). **(C)** Spread of the malaria-refractory trait through the female mosquito population, and consequences for mosquito and human infection status. Following releases of the drive system at year three, the proportion of refractory female mosquitoes (solid red line) increases and the proportion of infectious mosquitoes (dotted light blue line) declines. As humans recover from infection and less develop new infections, the *P*. *falciparum* parasite rate (solid green line) declines until it reaches near undetectable levels by year five. **(D)** Human malaria incidence is halted by the beginning of year four.

In Fig 4, we display a potential visualization scheme produced in Python for the simulations described above. The code to produce this visualization is available at https://github.com/Chipdelmal/MoNeT/tree/master/DataAnalysis/v2 (note that MGDrivE 2 code does not depend on Python). Fig 4A displays the climatological time-series data—temperature in magenta and rainfall in blue—which were used to calculate time-varying adult mosquito mortality rate and larval carrying capacity, respectively. The total adult female population size averaged over 100 stochastic runs is shown in green. This is relatively consistent throughout the year due to moderate seasonal changes in temperature in the tropical climate of the Comoros and the presence of permanent breeding sites such as cisterns throughout the island; however population spikes are observed after significant rainfall. Fig 4B displays allele frequencies for adult female mosquitoes over the simulation period. After eight consecutive weekly releases of 50,000 male mosquitoes homozygous for both the Cas9 (C) and gRNA/refractory (H) alleles three years into the simulation, we see the C and H alleles accumulate to high post-release frequencies, and the H allele continue to spread to a higher frequency over the subsequent ~6 months while the H and C alleles regularly co-occur enabling drive to occur at the gRNA locus. The wild-type allele (W) at the gRNA locus is almost completely lost over this period, and both in-frame and out-of-frame resistant alleles (R and B, respectively) accumulate to a small yet significant extent. The C allele slowly declines in frequency following the releases due to a fitness cost; and beginning ~1 year after the releases, the H allele gradually declines in frequency as its fitness cost begins to outweigh its inheritance bias. The declines in C and H allele frequencies continue beyond the simulated timeframe, although not before the H allele has a chance to interfere with disease transmission.

### 3.3. Epidemiological dynamics

The split drive system we consider includes a malaria-refractory gene that results in complete inability of mosquitoes to become infected with the malaria parasite, whether present in either one or two allele copies. In Fig 4C, we depict the spread of the malaria-refractory trait through the female mosquito population, and the consequences this has for mosquito and human infection status. Prior to the release, we see that infection prevalence in humans (*P*. *falciparum* parasite rate, PfPR) is mildly seasonal, with the proportion of infected humans (solid green line) waxing and waning in response to the fluctuating mosquito population size (green line in Fig 4A). The proportion of infectious female mosquitoes (dotted light blue line) oscillates in synchrony with the proportion of infected humans; but at a much lower proportion due to the short mosquito lifespan and the fact that most mosquitoes die before the parasite completes its EIP. Following releases of the split drive system and refractory gene at year three, the proportion of refractory female mosquitoes (red line) increases and, consequently, the proportion of infectious mosquitoes declines. As humans recover from infection and less develop new infections, the PfPR declines until it reaches near undetectable levels by year five. Lastly, Fig 4D depicts human malaria incidence, measured as the number of new infections per 1,000 humans per day. Stochastic variation in this model output is more pronounced due to the small number of incident cases relative to the total population. Incidence is halted by the beginning of year four, but PfPR takes almost a year longer to approach zero as infected humans clear parasites.

## 4. Availability and future directions

MGDrivE 2 is available at https://CRAN.R-project.org/package=MGDrivE2. The source code is under the GPL3 License and is free for other groups to modify and extend as needed. Mathematical details of the model formulation are available in the S1 Text, and documentation for all MGDrivE 2 functions, including vignettes, are available at the project’s website at https://marshalllab.github.io/MGDrivE/docs_v2/index.html. To run the software, we recommend using R version 2.10 or higher.

We are continuing development of the MGDrivE 2 software package and welcome suggestions and requests from the research community regarding future directions. The field of gene drive research is moving quickly, and we intend the MGDrivE 2 framework to serve as a flexible tool to address exploratory, logistical and operational questions regarding genetics-based control systems for mosquito disease vectors. This includes exploratory modeling of novel genetic constructs, assessment of candidate constructs against TPPs and PPCs, and field trial planning as constructs progress through the development pipeline. Current functionality presents a new opportunity to explore modeling-based research topics such as the invasiveness of threshold-dependent drive systems in the presence of climate fluctuations, seasonal source-sink dynamics and evolution towards smaller fitness costs. Future functionality that we are planning includes: i) modeling of mosquito traps to address questions related to monitoring and surveillance, and ii) more detailed epidemiological models addressing phenomena important to malaria and arbovirus transmission—for instance, dengue models that incorporate multiple serotypes with temporary cross-protective immunity and complications related to antibody-dependent enhancement [40], and malaria models that incorporate age-structure, immunity, asymptomatic infection and superinfection [41].

Additionally, we are exploring numerical sampling algorithms that can increase computational efficiency and speed, facilitated by separation of model specification and simulation in the software. The complexity of models that can be developed in MGDrivE 2 means that sensitivity analyses can become extremely computationally intensive, and the ability of the SPN framework to leverage efficient algorithms in these circumstances will be highly valuable. We also continue to be interested in developing a corresponding individual-based model capable of efficient modeling when the number of possible states exceeds the number of individuals in the population—for instance, for multi-locus systems such as daisy-drive [42] and multiplexing schemes in which a single gene is targeted at multiple locations to reduce the rate of resistance allele formation [43], and for epidemiological models in which age structure, immunity and mosquito biting heterogeneity become prohibitive for population models [41].

As gene drive technology matures, potential species of interest are not limited to arthropod vectors of disease. In addition to public health applications, gene drive has been proposed as a technique to help address problems in agriculture and conservation, with target species including insect agricultural pests [44] and invasive rodents that predate native birds [45]. While MGDrivE 1 was not designed with non-arthropod species in mind, it has been adapted for application to invasive rodents on islands [46]. We expect MGDrivE 2 to be easier to adapt to other insect or mammalian species due to the separation of model specification from simulation, meaning there is no need to adapt code to numerically simulate trajectories. To adapt model specification, the set of places and transitions for the SPN will need to be updated according to the life stages and development and mortality rates for the species of interest. New SPN transitions may be needed for behaviors not currently included, such as multiple mating in adults, while redundant transitions may be removed.

## Supporting information

S1 Table Model parameters describing the gene drive construct, mosquito bionomics and malaria epidemiology for simulations resembling releases on Grand Comore, Union of the Comoros. (PDF)

S1 Text Description of the modeling framework. A description of the mathematical equations that govern the inheritance, life history, landscape and epidemiology modules and the stochastic Petri net model formulation.
(PDF)
