# Supplementary material for: MGDrivE 2: A simulation framework for gene drive systems incorporating seasonality and epidemiological dynamics
Source: PLoS Comput Biol. 2021 May 21;17(5):e1009030. doi: 10.1371/journal.pcbi.1009030 (PMC8186770; doi:10.1371/journal.pcbi.1009030)
Supplement: S1 Table — (PDF) [file pcbi.1009030.s001.pdf]

Table S1. Model parameters describing the gene drive construct, mosquito bionomics and malaria epidemiology for simulations resembling releases on Grand Comore, Union of the Comoros.

Parameter:	Symbol:	Value:	Reference:
**Gene drive construct:**			
Cleavage rate in females	$c_{H,F}$	0.788	[1]
Cleavage rate in males	$c_{H,M}$	0.480	[1]
Proportion of cleaved alleles subject to accurate homology-directed repair (HDR) in females	$p_{HDR,F}$	0.798	[1]
Proportion of cleaved alleles subject to accurate HDR in males	$p_{HDR,M}$	0.769	[1]
Proportion of resistance alleles that are in-frame, functional	p_{RES}	0.167	[1]
Reduction in adult lifespan for gRNA/refractory allele homozygotes	s_{HB}	0.1	[1]
Reduction in female fecundity per Cas9 allele	$s_{C,F}$	0.078	[1]
**Mosquito bionomics:**			
Egg production per adult female (day^{-1})	β	32	[2]
Mean duration of egg stage (days)	T_E	3	[3]
Mean duration of larval stage (days)	T_L	7	[3]
Mean duration of pupa stage (days)	T_P	2	[3]
Coefficient of variation (duration of egg stage)	$\mathrm{CV}(T_E)$	0.2	[4]
Coefficient of variation (duration of larval stage)	$\mathrm{CV}(T_L)$	0.3	[4]
Coefficient of variation (duration of pupa stage)	$\mathrm{CV}(T_P)$	0.2	[4]
Carrying capacity of environment (larvae)	K	(time-varying)	Data: ERA5, Method: [5]
Mortality rate of adult mosquitoes (day^{-1})	μ_F, μ_M	(time-varying)	Data: ERA5, Method: [6]

**Malaria transmission:**			
Blood feeding rate	f	1/3	[7]
Human blood index	Q	0.9	[7]
Transmission efficiency: infected mosquito to human	b	0.55	[7]
Transmission efficiency: infected human to mosquito	c	0.15	[7]
Mean duration of extrinsic incubation period (days)	EIP ($1/\gamma_V$)	10	[8]
Coefficient of variation of extrinsic incubation period	CV(EIP)	0.4	[9]
Human infectious period (days)	$1/r$	200	[7]
Human lifespan (years)	$1/\mu_H$	62	[10]
Human population size	N_H	350,998	[10]

References:

1. Li, M. *et al.* (2020) ‘Development of a confinable gene drive system in the human disease vector Aedes aegypti’, *eLife*, 9(2020): e51701.
2. Depinay, J.-M. O. *et al.* (2004) ‘A simulation model of African Anopheles ecology and population dynamics for the analysis of malaria transmission’, *Malaria journal*, 3, p. 29.
3. Yaro, A. S. *et al.* (2006) ‘The distribution of hatching time in Anopheles gambiae’, *Malaria journal*, 5, p. 19.
4. Bayoh, M. N. and Lindsay, S. W. (2003) ‘Effect of temperature on the development of the aquatic stages of Anopheles gambiae sensu stricto (Diptera: Culicidae)’, *Bulletin of entomological research*, 93(5), pp. 375–381.
5. White, M. T. *et al.* (2011) ‘Modelling the impact of vector control interventions on Anopheles gambiae population dynamics’, *Parasites & vectors*, 4, p. 153.
6. Mordecai, E. A. *et al.* (2019) ‘Thermal biology of mosquito-borne disease’, *Ecology letters*, 22(10), pp. 1690–1708.
7. Smith, D. L. and McKenzie, F. E. (2004) ‘Statics and dynamics of malaria infection in Anopheles mosquitoes’, *Malaria journal*, 3, p. 13.
8. Smith, D. L., Dushoff, J. and Ellis McKenzie, F. (2004) ‘The Risk of a Mosquito-Borne Infectionin a Heterogeneous Environment’, *PLoS Biology*, p. e368. doi: 10.1371/journal.pbio.0020368.
9. Huber, J. H. *et al.* (2016) ‘Quantitative, model-based estimates of variability in the generation and serial intervals of Plasmodium falciparum malaria’, *Malaria journal*, 15(1), p. 490.
10. INSEED (2015) *Annuaire Statistique des Comores. Moroni, Union of the Comoros*. National Institute of Statistics and Economic and Demographic Studies (INSEED).
