# Supplementary material for: MGDrivE 2: A simulation framework for gene drive systems incorporating seasonality and epidemiological dynamics
Source: PLoS Comput Biol. 2021 May 21;17(5):e1009030. doi: 10.1371/journal.pcbi.1009030 (PMC8186770; doi:10.1371/journal.pcbi.1009030)
Supplement: S1 Text — A description of the mathematical equations that govern the inheritance, life history, landscape and epidemiology modules and the stochastic Petri net model formulation. (PDF) [file pcbi.1009030.s002.pdf]

MGDrivE 2: Mosquito Gene Drive Explorer 2
S1 Text: Description of the Modeling Framework

Sean L. Wu[1], Jared B. Bennett[2], Héctor M. Sánchez C.[1], Andrew J. Dolgert[3], Tomás M. León[1], and John M. Marshall[1]

[1]Divisions of Biostatistics and Epidemiology, University of California, Berkeley
[2]Graduate Group in Biophysics, University of California, Berkeley
[3]Institute for Health Metrics and Evaluation, Washington, Seattle

May 8, 2021

1 Lifecycle Model

The lifecycle model is similar to the discrete time ecology module used in **MGDrivE** [1]. Major differences include the switch to continuous time and replacement of fixed, constant delays with Erlang distributed delays in aquatic life stages. This change means that, whereas **MGDrivE**'s deterministic model was formulated as a set of delay difference equations, **MGDrivE 2**'s deterministic model is a set of ordinary differential equations (ODEs) (using the "linear chain trick" to simulate Erlang-distributed delays, [2]).

Similar to **MGDrivE**, the lifecycle model includes egg (E), larval (L), and pupal (P) aquatic stages. Upon emergence from P, adult mosquitoes are assigned a sex, the probability of which may depend on genotype. Upon emergence, females (F) become mated in the presence of male mosquitoes (M), and oviposit at an age-independent (though possibly time-dependent) rate until they die. If there are no adult males, newly emerging females transfer to an unmated adult female (U) compartment, where they remain until death or successful mating if males become available.

The system of ODEs describing the deterministic lifecycle model are solved at their non-trivial equilbrium to provide initial conditions for simulations in **MGDrivE 2**. These ODEs are a limiting case of the stochastic continuous-time Markov chain (CTMC) model, when populations are large (for technical conditions, see [3]). In our presentation of the ODEs and their equilibrium solutions, we ignore indexing by genotype because, for most simulations, the equilibrium solution corresponds to a baseline scenario prior to releases of modified mosquitoes, where mosquito populations are composed solely of wild-types. These equations also ignore indexing by node. We use (n_E, n_L, n_P) to denote the number of sub-stages in each aquatic stage (the Erlang shape parameter). Subscript i refers to any particular sub-stage, such that eggs are denoted E_i, larvae as L_i, pupae as P_i, and N_F, N_M the mated adult female and male populations, respectively. Because the non-trivial equilibrium will have zero unmated females, there is no N_U compartment.

Shape and rate parameters for the Erlang-distributed delays can be constructed as follows. Consider a random delay with mean $\frac{1}{q}$ and variance $\frac{1}{nq^2}$, where n is an integer. Such a random delay can be assumed to follow an Erlang distribution, and one way to construct a model of this system is to build a linear system of n bins, where the rate of transfer from the $i-1^{th}$ to i^{th} bin is given by qn. In a deterministic model, this will be a linear system of ODEs and, for a stochastic model, a CTMC.

We present the life history model with two different parameterizations of larval density dependence, which we call the "Lotka-Volterra" and "Logistic" versions, in reference to ecological theory. Both sets of equations are available in the code, and are provided as an example of how to use different functional forms of rate equations with the same Petri net structure.

1.0.1 Lotka-Volterra Density-Dependent Equations

This set of equations uses a linear form of per-capita density-dependent mortality for the larval instar stages that corresponds to the functional form assumed by [4].

$$
\begin{aligned}
\frac{d}{dt}E_1 &= (\beta N_F) - (\mu_E + q_E n_E)\,E_1 \\
\frac{d}{dt}E_i &= q_E n_E E_{i-1} - (\mu_E + q_E n_E)\,E_i;\; i = (2, ..., n_E) \\
\frac{d}{dt}L_1 &= q_E n_E E_{n_E} - \left(\mu_L + \alpha \sum_j L_j + q_L n_L\right) L_1 \\
\frac{d}{dt}L_i &= q_L n_L L_{i-1} - \left(\mu_L + \alpha \sum_j L_j + q_L n_L\right) L_i;\; i = (2, ..., n_L) \\
\frac{d}{dt}P_1 &= q_L n_L L_{n_L} - (\mu_P + q_P n_P)\,P_1 \\
\frac{d}{dt}P_i &= q_P n_P P_{i-1} - (\mu_P + q_P n_P)\,P_i;\; i = (2, ..., n_P) \\
\frac{d}{dt}N_F &= \phi q_P n_P P_{n_P} - \mu_F N_F \\
\frac{d}{dt}N_M &= (1-\phi) q_P n_P P_{n_P} - \mu_M N_M
\end{aligned}
\tag{1}
$$

In this set of equations, the parameter α represents increased mortality rates that occur as a function of crowding, and has units of $\text{time}^{-1}\text{area}^{2}$.

To solve the model with linear density-dependence at equilibrium, we assume that the equilibrium number of adult female mosquitoes, $\overline{N_F}$ is known, and that all rate constants, with the exception of α are also fixed; we then solve for all remaining state variables plus α, giving a system of $2 + n_E + n_L + n_P$ equations and the same number of unknowns.

$$
\begin{aligned}
\overline{P_{n_P}} &= \frac{\overline{N_F}\mu_F}{n_P q_P \phi} \\
\overline{N_M} &= \frac{(1-\phi) n_P q_P \overline{P_{n_P}}}{\mu_M} \\
\overline{P_i} &= \left(\frac{\mu_P + q_P n_P}{q_P n_P}\right) \overline{P_{i+1}};\ i = (n_P - 1, ..., 1) \\
\overline{E_1} &= \frac{\beta \overline{N_F}}{\mu_E + q_E n_E} \\
\overline{E_i} &= \left(\frac{q_E n_E}{\mu_E + q_E n_E}\right) \overline{E_{i-1}};\ i = (2, ..., n_E) \\
\overline{L_{n_L}} &= \left(\frac{\mu_P + q_P n_P}{q_L n_L}\right) \overline{P_1} \\
\overline{L_1} &= \frac{\overline{E_{nE}}^{\left(\frac{n_L - 1}{n_L}\right)} \overline{L_{nL}}^{\left(\frac{1}{n_L}\right)} \underline{n_E}^{\left(\frac{n_L-1}{n_L}\right)} \underline{q_E}^{\left(\frac{n_L-1}{n_L}\right)}}{\underline{n_L}^{\left(\frac{n_L-1}{n_L}\right)} \underline{q_L}^{\left(\frac{n_L-1}{n_L}\right)}} \\
\overline{L_i} &= \overline{L_1}^{i} \left(\frac{n_L q_L}{\overline{\overline{E_{nE}}} n_E q_E}\right)^{i-1};\ i = (2, ..., n_L - 1) \\
\alpha &= \frac{\frac{\overline{E_{nE}} n_E q_E}{\overline{L_1}} - (\mu_L + n_L q_L)}{\sum_j \overline{L_j}}
\end{aligned}
\tag{2}
$$

1.0.2 Logistic Density-Dependent Equations

This set of equations uses a rational form of per-capita density-dependent mortality for the larval instar stages that uses a carrying capacity K parameterization (equivalent to the logistic model in ecology).

$$
\begin{aligned}
\frac{d}{dt}E_1 &= (\beta N_F) - (\mu_E + q_E n_E) E_1 \\
\frac{d}{dt}E_i &= q_E n_E E_{i-1} - (\mu_E + q_E n_E) E_i;\ i = (2, ..., n_E) \\
\frac{d}{dt}L_1 &= q_E n_E E_{n_E} - \left(\mu_L \left(1 + \frac{\sum_j L_j}{K}\right) + q_L n_L\right) L_1 \\
\frac{d}{dt}L_i &= q_L n_L L_{i-1} - \left(\mu_L \left(1 + \frac{\sum_j L_j}{K}\right) + q_L n_L\right) L_i;\ i = (2, ..., n_L) \\
\frac{d}{dt}P_1 &= q_L n_L L_{n_L} - (\mu_P + q_P n_P) P_1 \\
\frac{d}{dt}P_i &= q_P n_P P_{i-1} - (\mu_P + q_P n_P) P_i;\ i = (2, ..., n_P) \\
\frac{d}{dt}N_F &= \phi q_P n_P P_{n_P} - \mu_F N_F \\
\frac{d}{dt}N_M &= (1-\phi) q_P n_P P_{n_P} - \mu_M N_M
\end{aligned}
\tag{3}
$$

In this parameterization of density-dependent mortality, μ_L is the natural mortality rate of larvae without any effects of resource depletion or competition (because when $\sum_j L_j$ is small, the mortality is approximately

μ_L).

To solve the model at equilibrium, we assume that the equilibrium number of adult female mosquitoes, $\overline{N_F}$ is known, and that all rate constants, with the exception of K, are also fixed; we then solve for all remaining state variables plus K, giving a system of $2 + n_E + n_L + n_P$ equations and the same number of unknowns.

$$
\begin{aligned}
\overline{P_{n_P}} &= \frac{\overline{N_F}\mu_F}{n_P q_P \phi} \\
\overline{N_M} &= \frac{(1-\phi) n_P q_P \overline{P_{n_P}}}{\mu_M} \\
\overline{P_i} &= \left(\frac{\mu_P + q_P n_P}{q_P n_P}\right) \overline{P_{i+1}};\ i = (n_P - 1, ..., 1) \\
\overline{E_1} &= \frac{\beta \overline{N_F}}{\mu_E + q_E n_E} \\
\overline{E_i} &= \left(\frac{q_E n_E}{\mu_E + q_E n_E}\right) \overline{E_{i-1}};\ i = (2, ..., n_E) \\
\overline{L_{n_L}} &= \left(\frac{\mu_P + q_P n_P}{q_L n_L}\right) \overline{P_1} \\
\overline{L_1} &= \frac{\overline{E_{nE}}^{\left(\frac{n_L - 1}{n_L}\right)} \overline{L_{nL}}^{\left(\frac{1}{n_L}\right)} {n_E}^{\left(\frac{n_L - 1}{n_L}\right)} {q_E}^{\left(\frac{n_L - 1}{n_L}\right)}}{{n_L}^{\left(\frac{n_L - 1}{n_L}\right)} {q_L}^{\left(\frac{n_L - 1}{n_L}\right)}} \\
\overline{L_i} &= \frac{\overline{L_{n_L}}^{(\frac{i}{n_L})} \overline{E_{n_E}}^{(\frac{n_L - i}{n_L})} {n_E}^{(\frac{n_L - i}{n_L})} {q_E}^{(\frac{n_L - i}{n_L})}}{{n_L}^{(\frac{n_L - i}{n_L})} {q_L}^{(\frac{n_L - i}{n_L})}};\ i = (1, ..., n_L - 1) \\
K &= \frac{\sum_j \overline{L_j}}{\left(\frac{q_E n_E \overline{E_{n_E}}}{\mu_L \overline{L_1}} - \frac{q_L n_L}{\mu_L} - 1\right)}
\end{aligned}
\tag{4}
$$

In fact, because both of these per-capita density dependent rates of mortality are linear functions in the number of larvae present (such that the overall mortality is quadratic in the number of larvae), at equilibrium the parameters α and K are related by the simple expression:

$$
\frac{\mu_L}{K} = \alpha \tag{5}
$$

1.1 Parameters

Due to the continuous-time model structure as well as reformulating the fixed delays of **MGDrivE** as Erlang-distributed random delays, parameters used in **MGDrivE** cannot be directly "plugged-in" to **MGDrivE 2** simulations. In this section we discuss how to parameterize **MGDrivE 2**, and discuss similarities and differences with those in [1, 5]. We note that there will be certain mathematical artifacts which prevent a one-to-one mapping between the two models due to the change between a lagged discrete-time Markov chain (DTMC) to continuous-time Markov chain (CTMC) model formulation. For more details on how these arise and their effects, please consult [6, 7].

1.1.1 Aquatic Survival

Let the probability to survive any aquatic state $x \in \{E, L, P\}$ be θ_x. In **MGDrivE**, these were given as:

$$\theta_x = (1 - \mu_x)^{T_x} \tag{6}$$

In **MGDrivE 2**, the aquatic state is broken in n_x substages to produce an overall Erlang-distributed dwell time, τ. The Erlang distribution has shape parameter n_x and rate parameter $n_x q_x$, where $q_x = \frac{1}{T_x}$; therefore $\mathbb{E}[\tau] = \frac{1}{q_x} = T_x$ and $\text{Var}[\tau] = \frac{1}{n_x q_x^2}$. Because the dwell time τ is a random variable the probability of survival is expressed :

$$\begin{aligned}
\theta_x &= \int_0^\infty e^{-\mu_x \tau} \text{ Erlang}(\tau; n_x, q_x n_x) d\tau \\
\theta_x &= \int_0^\infty e^{-\mu_x \tau} \frac{q_x n_x^{n_x}}{(n_x - 1)!} \tau^{n_x - 1} e^{-q_x n_x \tau} d\tau \\
&= \left(\frac{q_x n_x}{q_x n_x + \mu_x} \right)^{n_x}
\end{aligned} \tag{7}$$

If we wanted to match survival probabilities between the two models, we just consider μ_x in equation 7 to be an unknown and solve for it:

$$\mu_x = \frac{q_x n_x}{\sqrt[n_x]{\theta_x}} - q_x n_x \tag{8}$$

Note that we can arrive at the solution from equation 7 by considering not a single random variable τ but rather the random variables X, Y, where the latter is the time to death, if death were to occur, and the former is time to advancement out of stage x, were advancement to occur. Then we want the probability that $X < Y$:

$$\begin{aligned}
P(X < Y) &= \int_0^\infty \int_0^\tau \mu_x e^{-\mu_x \tau} \text{ Erlang}(\tau'; n_x, q_x n_x)\, d\tau d\tau' \\
&= \left(\frac{q_x n_x}{q_x n_x + \mu_x} \right)^{n_x}
\end{aligned} \tag{9}$$

Intuition behind the solution may be acquired if we take literally the interpretation of the Erlang distribution as being used in the "linear chain trick"; in this case at each substage the overall probability of survival is $\left(\frac{q_x n_x}{q_x n_x + \mu_x} \right)$. Because there are n_x substages, the total survival probability is the product of the n_x stages.

1.1.2 Population Growth Rate

In **MGDrivE**, the intrinsic population growth rate R_m was defined as "equal to the rate of female egg production multiplied by the life expectancy of an adult mosquito multiplied by the proportion of eggs that will survive through all of the juvenile life stages in the absence of density-dependence" [5].

In **MGDrivE** it had units of mosquito^{-1}day^{-1}:

$$R_M = \left(\frac{\beta}{\mu}\right)\left(\theta_E \theta_L \theta_P (1-\mu)\left(\frac{1}{2}\right)\right) \tag{10}$$

It is essentially the same in **MGDrivE 2**, using the form of θ_x from equation 6:

$$R_M = \left(\frac{\beta}{\mu}\right)(\theta_E \theta_L \theta_P \phi) \tag{11}$$

Note however, the absence of the $1-\mu$ term; this is because equation 11 is a continuous time rate; adults are available to oviposit immediately upon emergence, so there is no need extra mortality between emergence and adulthood [8].

1.1.3 Parameterization from Growth Rates

In **MGDrivE** the model was typically parameterized such that equilibrium solutions were available in closed form. The assumptions, outlined in the supplemental information of [5] and based on the model of [8], are that $\mu_E = \mu_L = \mu_P$; that is, the density-independent mortality of each aquatic stage is the same. In the absence of density-dependent effects, the total probability of surviving the aquatic stages was $(1-\mu_L)^{(T_E+T_L+T_P)}$. Combined with knowledge of the generation time g, the daily population (geometric) growth rate r_M, and the per-generation geometric growth $R_M = (r_M)^g$, μ_L could be found in closed form, and from that the remaining unknowns, α and L_{eq} (strength of density dependence and equilibrium larval population) could also be solved in closed form. The relevant equations were S51-S55 in [1].

In **MGDrivE 2** we want to be able to solve for equilibria under similar assumptions of equal density-independent mortality across aquatic stages. Let us define $\mu_A = \mu_E = \mu_L = \mu_P$ so we seek a solution to the unknown constant aquatic stage mortality μ_A. We first expand Equation 11 in terms of Equation 7:

$$R_M = \left(\frac{\beta}{\mu}\right)\left(\frac{q_E n_E}{q_E n_E + \mu_A}\right)^{n_E}\left(\frac{q_L n_L}{q_L n_L + \mu_A}\right)^{n_L}\left(\frac{q_P n_P}{q_P n_P + \mu_A}\right)^{n_P}\phi \tag{12}$$

Here, R_M is the per-generation growth rate; in **MGDrivE** it was $(r_M)^g$. However, because **MGDrivE 2** is a continuous time model, the equations for geometric growth are not appropriate. The equivalent infinitesimal rate of growth is $\log(r_M)$ such that $R_M = e^{g\log(r_M)}$, which is the left hand side of Equation 12.

Finding closed form solutions to this equation is difficult because of the additional terms introduced by the Erlang delays. Therefore we use Newton's method in **R** (with `uniroot`) to numerically solve for μ_A. The input to the function is the daily growth rate r_M and biological parameters $\beta, \mu, q_E, n_E, q_L, n_L, q_P, n_P, \phi$, and it returns the value of μ_A such that the following equation holds:

$$\left(e^{\log(r_M)g}\right) - \left[\phi\left(\frac{\beta}{\mu}\right)\left(\frac{q_E n_E}{q_E n_E + \mu_A}\right)^{n_E}\left(\frac{q_L n_L}{q_L n_L + \mu_A}\right)^{n_L}\left(\frac{q_P n_P}{q_P n_P + \mu_A}\right)^{n_P}\right] = 0 \tag{13}$$

1.1.4 Lifespan Modification

In **MGDrivE**, the genotype-specific parameter ω was used to reduce lifespans of non wild-type organisms due to fitness costs associated with the homing cassette, or intentional fitness reduction. However, for **MGDrivE**

2 we generalize this to modify lifespans in either direction, relative to wildtype, because experimental data showed evidence of in some cases substantial lifespan *increases* from driving certain genetic material in model organisms [9].

Because wildtype lifespans (x) were geometrically distributed random variables, and daily survival was given by $(1-\mu)\omega$, one could solve for a reduced lifespan, $y < x$, by noting the daily mortality probability can be written $p = 1 - ((1-\mu)\omega) = 1 - \omega + \mu\omega$. Then note that the mean lifespan is $y = \frac{1}{1-\omega+\mu\omega}$. Thus to solve for ω, we solve for the root of the equation $\frac{1}{1-\omega+\mu\omega} - y = 0$, where only ω is unknown.

In **MGDrivE 2** adult lifespans are exponentially distributed random variables. To change the lifespan y (no longer restricted to $y < x$, y may be any positive number), consider modifying the mortality hazard by the factor ω. To find ω we just solve the following equation:

$$\begin{aligned} y &= \int_0^\infty \tau \; \mu\omega e^{-(\mu\omega)\tau} d\tau \\ \omega &= \frac{1}{y\mu} \end{aligned} \tag{14}$$

1.1.5 Movement

In the continuous time model, mosquito movement is given by a rate of movement from each node i to all other nodes $j \neq i$. When parameterizing these rates, we need to take into account that they will be a function of the total probability of a mosquito to leave its natal habitat i over its lifetime, P. Given that adult mosquitoes are subject to mortality with rate μ, one can solve for the rate of movement out of node i to anywhere (δ) as follows:

$$\begin{aligned} P &= \int_0^\infty \delta e^{-(\delta+\mu)\tau} d\tau \\ \delta &= \frac{\mu P}{1-P} \end{aligned} \tag{15}$$

Then, if from node i, we have some vector of movement probabilities $\{\pi_{ij}\}_{j\neq i}$ which give the probability to move from i to j conditional on leaving i, we can set the movement hazard between to be $\delta\pi_{ij}$ so we get the right lifetime leaving probability. The new vector of movement *hazards* is $\{\delta\pi_{ij}\}_{j\neq i}$.

1.2 Genetic Inheritance & Modification

Because **MGDrivE 2** builds upon our previous work [1], the data structure used in **MGDrivE** to store all probabilities, fitness costs, etc. related to genotypes (the "inheritance cube") is compatible with **MGDrivE 2**. In fact, we use the same cubes developed in that model and do not introduce any new cubes in this text.

While data structures can be reused, the fitness modifiers that describe the effect of inherited genotype on the life-history have a slightly different interpretation. In **MGDrivE**, genotype specific multipliers applied to daily probabilities, they had to be bounded in order to prevent nonsensical parameter values. If P represents a wild-type daily survival probability, for example, the modifier ω must be $0 < \omega < \frac{1}{P}$. Because **MGDrivE 2** is parameterized directly in terms of hazards, the genotype-specific effects can be any positive real number. This has the added benefit of making them amenable to parameterization directly from survival analysis routinely preformed on biological lab experiments which often estimate relative hazards [9].

2 Epidemiological Dynamics

To introduce epidemiological dynamics in **MGDrivE 2**, we use the SEI-SIS coupled model of mosquito and human infection dynamics as the basic model (Figure S1), as more complex vector-host models tend to be modifications of the basic form. This type of model is known as the Ross-Macdonald model in mathematical epidemiology [10, 11]. **MGDrivE 2** also supports SEI-SEIR models, which we introduce briefly later.

Here S_H refers to susceptible humans, I_H to infected/infectious humans, S_V to susceptible mosquitoes, $(E_{V,1}, ..., E_{V,n})$ to incubating mosquitoes, and I_V to infectious mosquitoes. Because only mated adult females undergo gonotrophic cycles which require bloodfeeding, infection dynamics are only present in F.

To investigate the dynamics of the model, it is important to focus on events (transitions) that change state, and the rate at which they occur. For epidemiological dynamics, the two primary events are mosquito to human transmission and human to mosquito transmission, each driven by a Poisson process. Computation of the rate with which each process occurs in time depends on the per-capita force of infection (FOI) terms: λ_H and λ_V, the rates at which any particular susceptible human gets infected and moves to the infected class, and the rate at which any particular susceptible mosquito gets infected and moves to the incubating class, respectively. When multiplied by the total numbers of susceptible humans or susceptible mosquitoes, respectively, we arrive at the correct rate for the Poisson processes.

Figure S1: SEI-SIS pathogen transmission system; orange arrows denote the contribution of each species to the force of infection term on the other.

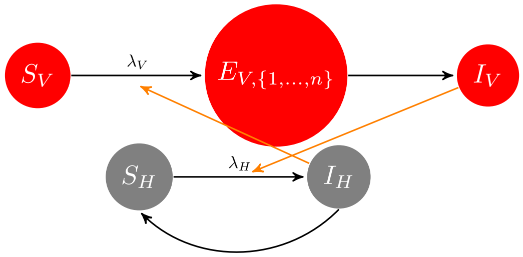

2.1 Transmission Terms

The function λ_H is the per-capita FOI on susceptible humans, such that $\lambda_H S_H$ is the total rate at which infection in the human population occurs in the deterministic model, or the intensity of the Poisson process for human infections in the stochastic model. Using Ross-Macdonald parameters as in [11], $\lambda_H = abI_V \left(\frac{1}{N_H}\right)$. This is because, if a is the human biting rate, b mosquito-human transmission efficiency, then the total number of infectious bites produced by the mosquito population is abI_V. Assuming uniform biting on humans, any particular person has probability $\frac{1}{N_H}$ of being bitten, so $abI_V \left(\frac{1}{N_H}\right)$ is the per-capita FOI. Multiplication by S_H, the total number of humans, gives the total rate of infection in the human population.

The per-capita FOI in the mosquito population is $\lambda_V S_V$. The FOI on susceptible mosquitoes is written as $\lambda_V = ac\left(\frac{I_H}{N_H}\right)$. Again, a is the human biting rate, c is the human-mosquito transmission efficiency, and $\frac{I_H}{N_H}$ is the probability that a bite lands on an infectious human. Multiplying by the total susceptible vector population, S_V, gives the total rate of infection in the mosquito population.

2.2 Deterministic Approximation

In this section we describe how to develop a mean-field approximation given by a system of ODEs to the stochastic SEI-SIS system.

2.2.1 Mean-field Approximation of Human Stochastic Dynamics

Here we describe in detail the method used to approximate the stochastic CTMC model of infection dynamics in the human population, as its state space is smaller and the same methods can be used for the larger mosquito dynamics. The methods follow those presented in [12]. Because we are only considering the human population, we drop the H superscript on state variables.

If we consider the mosquito population to be constant, as it would be at dynamic equilibrium for the deterministic model, then λ_H will be a constant and we can decouple the human SIS dynamics from the mosquito SEI system. We show a diagram of the human only dynamics in Figure S2.

Figure S2: Susceptible-infected-susceptible (SIS) human infection dynamics

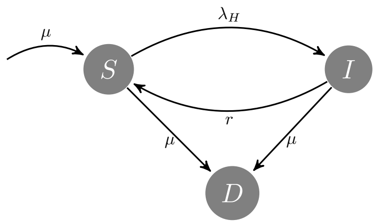

One way to analyze a CTMC is to derive the *Chapman-Kolmogorov equations* of the process. These equations give the conditional probabilities to transition to any ending state from a given starting state, over some time interval. For example if at time $t \leq t'$ the process (represented by $X(t)$) is in a state x, then the probability to jump to any state x' is $\mathbf{P}\left(X(t+t') = x' | X(t) = x\right)$, where $\mathbf{P}$ is a distribution over future states. It is often easier to work with the differential form of these equations, where the derivative is taken with respect to time, giving a system of differential equations that describes the time evolution of the Markov transition kernel over state space. Taking the derivative of these equations will involve expanding $\mathbf{P}(t+\delta t)$, which if expanded as $\mathbf{P}(t)\mathbf{P}(\delta t)$, leads to the linear system of ODEs known as the Kolmogorov forward equations (KFE).

The CTMC is a process which jumps between points in state space $(S, I) \to (S', I')$. Put another way, the joint density must be understood as giving the probability to transition between all unique pairs of ways a number of people can be either susceptible or infectious, when birth, death, infection, and recovery can change state. Taking into account all events that can cause the system state at time t to change, we can derive the KFE as:

$$
\begin{aligned}
\frac{d}{dt}\mathbf{P}(S, I; t) = & -\big(\mu(S+I) + \mu S + \mu I + \lambda_H S - rI\big)\,\mathbf{P}(S, I; t) \\
& + \mu\big((S-1) + I\big)\,\mathbf{P}(S-1, I; t) \\
& + \mu(S+1)\,\mathbf{P}(S+1, I; t) \\
& + \mu(I+1)\,\mathbf{P}(S, I+1; t) \\
& + \lambda_H(S+1\,)\mathbf{P}(S+1, I-1; t) \\
& + r(I+1)\,\mathbf{P}(S-1, I+1; t)
\end{aligned}
\tag{16}
$$

The KFE can be manipulated to derive the deterministic approximation to the CTMC. Because we assume constant λ_H, the hazard/rate functions for each event are all first order in the state variables, the deterministic approximation will accurately describe the expected value of the stochastic model.

To go about this, we first construct the *stoichiometry matrix* $\mathbf{S}_{u \times v}$, where u is dimension of the state space, and v is the number of unique events in the process. As the dimension of both state and event spaces are small, we can easily write this as:

$$\mathbf{S} = \begin{array}{c} \\ S \\ I \end{array} \begin{array}{c} \begin{array}{ccccc} \to S & S\to D & I \to D & S \to I & I \to S \end{array} \\ \left[\begin{array}{ccccc} 1 & -1 & 0 & -1 & 1 \\ 0 & 0 & -1 & 1 & -1 \end{array} \right] \end{array} \tag{17}$$

To begin developing our deterministic approximation, we take the time-derivative of the expectation of our state vector at time t, denoted as $X(t)$ (full derivation in [12]). We also define $h(X(t))$ as a v-dimensional column vector of rates/intensities of each event at that point x in state space.

$$\begin{aligned} \frac{d}{dt}\mathbb{E}\left[X(t)\right] &= \frac{d}{dt}\sum_{x\in\mathcal{M}} x\,\mathbf{P}(x;t), \text{ where } \mathcal{M} \text{ is the set of all allowed model states} \\ &= \sum_{x\in\mathcal{M}} x\,\frac{d}{dt}\mathbf{P}(x;t) \\ &= \sum_{x\in\mathcal{M}} x \sum_{i=1}^{v} \Big[h_i(x-\mathbf{S}^{(i)})\mathbf{P}(x-\mathbf{S}^i;t) + h_i(x)\mathbf{P}(x;t)\Big] \\ &\vdots \\ &= \sum_{i=1}^{v}\mathbb{E}\left[\mathbf{S}^{(i)}h_i\left(X(t)\right)\right] \\ &= \sum_{i=1}^{v}\mathbf{S}^{(i)}\mathbb{E}\left[h_i\left(X(t)\right)\right] \end{aligned} \tag{18}$$

After making the substitution $y(t) = \mathbb{E}(X(t))$, the above equation can be recognized as a matrix ODE giving the deterministic approximation of the system.

$$\begin{aligned} \frac{d}{dt}y(t) &= \sum_{i=1}^{v}\mathbf{S}^i h_i(y(t)) \\ &= \mathbf{S}\big(h(y(t))\big) \end{aligned} \tag{19}$$

Substituting in our stoichiometry matrix $\mathbf{S}$ and SIS hazard functions, we derive the following matrix ODE (writing out the column vector $y(t)$ explicitly in terms of our two state variables):

$$\frac{d}{dt}\begin{vmatrix} S(t) \\ I(t) \end{vmatrix} = \begin{vmatrix} 1 & -1 & 0 & -1 & 1 \\ 0 & 0 & -1 & 1 & -1 \end{vmatrix} \begin{vmatrix} \mu\big(S(t)+I(t)\big) \\ \mu S(t) \\ \mu I(t) \\ \lambda_H S(t) \\ rI(t) \end{vmatrix} \tag{20}$$

Separating the variables and completing the matrix vector multiplication leads to the familiar ODE form

of the SIS model with demography. We defer the equilibrium solution until later, when we can solve for the mosquito equilibrium jointly.

$$
\begin{aligned}
\frac{d}{dt}S(t) &= \mu\big(S(t)+I(t)\big) - \mu S(t) - \lambda_H S(t) + rI(t) \\
\frac{d}{dt}I(t) &= -\mu I(t) + \lambda_H S(t) - rI(t)
\end{aligned}
\tag{21}
$$

2.2.2 Mean-field Approximation of Mosquito Stochastic Dynamics

Similar as we did for humans, we consider λ_V to be a constant and decouple the mosquito SEI dynamics from the human SIS dyanmics. A flow graph of the mosquito dynamics is shown in Figure S3, which shows the possible states a adult female mosquito may exist in during its life, with death (D) as an absorbing state. As described in the main text, we partition the exposure (extrinsic incubation period, EIP) into n compartments such that the overall dwell time is Erlang distributed. As before, because we are purely focused on the mosquito model, we drop the V superscript for state variables.

For a single adult female mosquito, the transition rates on the edges of the graph specify the hazard rates of leaving the current state; the aggregated process for a population of mosquitoes sums the individual hazards by the number of mosquitoes in that state.

Figure S3: Susceptible-exposed-infected (SEI) mosquito infection dynamics

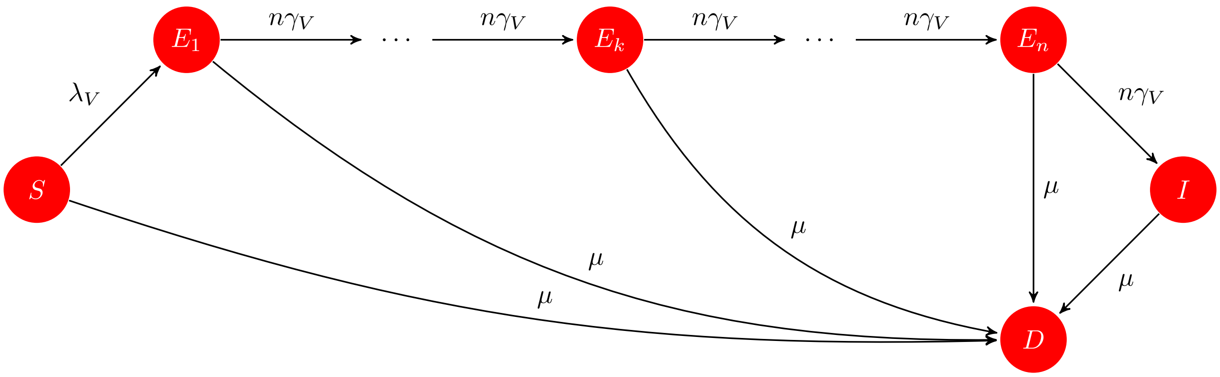

Upon emergence, the mosquito enters the susceptible S state, subject to force of infection λ_V. Susceptible mosquitoes are also subject to a mortality rate, μ, which is constant across all compartments, leading to an exponentially distributed lifespan with mean $\frac{1}{\mu}$. If the mosquito becomes infected, which occurs at rate λ_V, it will advance through the extrinsic incubation period (EIP) prior to becoming infectious.

The EIP is broken into n bins, with transition from the kth to $k+1^{\text{th}}$ occurring at a rate nq. This specification allows an Erlang (Gamma with integer shape parameter) distributed duration of EIP, with mean $\frac{1}{\gamma_V}$ and variance $\frac{1}{n\gamma_V^2}$. A mosquito survives the EIP with probability $\lim_{n\to\infty}(\frac{n\gamma_V}{n\gamma_V+\mu})^n = e^{-\frac{\mu}{\gamma_V}}$. Conditional on survival, the proportion of mosquitoes that become infectious t days after becoming infected is distributed as $\text{Gamma}(t; n, \frac{1}{n\gamma_V}) = \frac{(n\gamma_V)^n}{(n-1)!}t^{n-1}e^{-tn\gamma_V}$ (using shape/scale parameterization).

Written in matrix form, the infinitesimal generator matrix for a single adult female mosquito, or a cohort emerging at the same time has the following form:

$$
\mathbf{Q} = \begin{array}{c} \\ S \\ E_1 \\ \vdots \\ E_k \\ \vdots \\ E_n \\ I \\ D \end{array}
\begin{array}{c}
\begin{array}{cccccccc} S & E_1 & \cdots & E_k & \cdots & E_n & I & D \end{array} \\
\begin{bmatrix}
-(\lambda_V+\mu) & \lambda_V & \dots & 0 & \dots & 0 & 0 & \mu \\
0 & -(n\gamma_V+\mu) & n\gamma_V & 0 & \dots & 0 & 0 & \mu \\
 & & & & & & & \\
0 & 0 & \dots & -(n\gamma_V+\mu) & n\gamma_V & 0 & 0 & \mu \\
 & & & & & & & \\
0 & 0 & \dots & 0 & \dots & -(n\gamma_V+\mu) & n\gamma_V & \mu \\
0 & 0 & \dots & 0 & \dots & 0 & -\mu & \mu \\
0 & 0 & \dots & 0 & \dots & 0 & 0 & 0
\end{bmatrix}
\end{array}
\tag{22}
$$

For the model of a single adult female mosquito, or a cohort that emerged at the same time, the infinitesimal generator (Equation 22), the KFE is:

$$
\begin{aligned}
\frac{d}{dt}\mathbf{P}(S;t) &= -\lambda_V S\mathbf{P}(S;t) - \mu\mathbf{P}(S;t) \\
\frac{d}{dt}\mathbf{P}(E_1;t) &= \lambda_V S\mathbf{P}(S;t) - n\gamma_V\mathbf{P}(E_1;t) - \mu\mathbf{P}(E_1;t) \\
&\vdots \\
\frac{d}{dt}\mathbf{P}(E_k;t) &= n\gamma_V\mathbf{P}(E_{k-1};t) - n\gamma_V\mathbf{P}(E_k;t) - \mu\mathbf{P}(E_k;t) \\
&\vdots \\
\frac{d}{dt}\mathbf{P}(E_n;t) &= n\gamma_V\mathbf{P}(E_{n-1};t) - n\gamma_V\mathbf{P}(E_n;t) - \mu\mathbf{P}(E_n;t) \\
\frac{d}{dt}\mathbf{P}(I;t) &= n\gamma_V\mathbf{P}(E_n;t) - \mu\mathbf{P}(I;t) \\
\frac{d}{dt}\mathbf{P}(D;t) &= \mu\big(\mathbf{P}(S;t) + \mathbf{P}(E_1;t) + \ldots + \mathbf{P}(E_n;t) + \mathbf{P}(I;t)\big)
\end{aligned}
\tag{23}
$$

While Equation 23 describes how the probability distribution over states for a cohort of mosquitoes changes over time, to account for emergence (which we will need for the deterministic approximation), we let ϵ give the rate at which females emerge into S from pupae. For brevity, $\mathbf{P}(\ldots;t)$ appearing in the joint density function means that those elements of the random vector do not change.

$$
\begin{aligned}
\frac{d}{dt}\mathbf{P}(S,E_1,\ldots,E_n,I;t) = {} & \epsilon\,\mathbf{P}(S-1,\ldots;t) - \epsilon\,\mathbf{P}(\ldots;t) \\
& - \lambda_V S\mathbf{P}(\ldots;t) + \lambda_V(S+1)\mathbf{P}(S+1,E-1,\ldots;t) \\
& - (2+n)\mu\mathbf{P}(\ldots;t) \\
& + \mu\mathbf{P}(S+1,\ldots;t) + \mu\mathbf{P}(\ldots,E_1+1,\ldots;t) + \ldots + \mu\mathbf{P}(\ldots,I+1;t) \\
& - (n\gamma_V)n\mathbf{P}(\ldots;t) \\
& + qn\mathbf{P}(\ldots,E_1+1,E_2-1,\ldots;t) + \ldots + n\gamma_V\mathbf{P}(\ldots,E_n+1,I-1;t)
\end{aligned}
\tag{24}
$$

As we did for deriving the deterministic approximation of human infection dynamics, we write out the stoichiometry matrix $\mathbf{S}_{u\times v}$ of dimensions $(2+n)\times(2n+4)$. Because each column in $\mathbf{S}$ describes an allowable jump in state space, the total number of terms in the KFEs should be equal to $2(2n+4) = 4n+8$; checking this with the derivation in the previous section allows us to confirm that the equations are correct. Once we have $\mathbf{S}$, the mean-field approximation follows the same method as in Section 2.2.1.

$$
\mathbf{S} = \begin{array}{c} \\ S \\ E_1 \\ \vdots \\ E_i \\ \vdots \\ E_n \\ I \end{array}
\begin{array}{c}
\begin{array}{ccccccccccc} \to S & S\to E_1 & S\to D & E_1\to E_2 & E_1\to D & \dots & E_i\to E_{i+1} & E_i\to D & \dots & E_n\to I & E_n\to D & I\to D \end{array} \\
\begin{bmatrix}
1 & -1 & 0 & 0 & 0 & \dots & 0 & 0 & \dots & 0 & 0 & 0 \\
0 & 1 & -1 & -1 & -1 & \dots & 0 & 0 & \dots & 0 & 0 & 0 \\
\vdots & \vdots & \vdots & \vdots & \vdots & \vdots & \vdots & \vdots & \vdots & \vdots & \vdots & \vdots \\
0 & 0 & 0 & 0 & 0 & \dots & -1 & -1 & \dots & 0 & 0 & 0 \\
\vdots & \vdots & \vdots & \vdots & \vdots & \vdots & \vdots & \vdots & \vdots & \vdots & \vdots & \vdots \\
0 & 0 & 0 & 0 & 0 & \dots & 0 & 0 & \dots & -1 & -1 & 0 \\
0 & 0 & 0 & 0 & 0 & \dots & 0 & 0 & \dots & 1 & 0 & -1
\end{bmatrix}
\end{array} \tag{25}
$$

Using the stoichiometry and the KFEs we write down the approximating equations in matrix ODE form:

$$
\frac{d}{dt} \begin{vmatrix} S(t) \\ E_1(t) \\ \vdots \\ E_n(t) \\ I(t) \end{vmatrix} = \mathbf{S} \begin{vmatrix} \epsilon \\ \lambda_V S(t) \\ \mu S(t) \\ \vdots \\ n\gamma_V E_n(t) \\ \mu E_n(t) \\ \mu I(t) \end{vmatrix} \tag{26}
$$

Because the emergence rate ϵ and the force of infection on mosquitoes λ_V are considered a constants, all jump terms are of zero or first order and the deterministic approximation will correctly approximate the mean behavior of the stochastic system. For clarity, we write the system of linear ODEs component-wise:

$$
\begin{aligned}
&\frac{d}{dt}S(t) = \epsilon - \lambda_V S(t) - \mu S(t) \\
&\frac{d}{dt}E_1(t) = \lambda_V S(t) - n\gamma_V E_1(t) - \mu E_1(t) \\
&\quad\vdots \\
&\frac{d}{dt}E_n(t) = n\gamma_V E_{n-1}(t) - n\gamma_V E_n(t) - \mu E_n(t) \\
&\frac{d}{dt}I(t) = n\gamma_V E_n(t) - \mu I(t)
\end{aligned} \tag{27}
$$

2.3 Quasi-stationary distribution for mosquito infection dynamics

In order to solve the coupled mosquito SEI - human SIS model at equilibrium, we need to be able to solve for the distribution of adult female mosquitoes across states $(S, E_1, ..., E_n, I)$. This is because, given an endemic equilibrium prevalence in humans, we can compute the number of infectious mosquitoes I required to sustain that prevalence of disease. From that, we can use the quasi-stationary solution of the CTMC model given in Equation 22 to compute the total adult female mosquito population and their distribution across stages, which can then be plugged into the life history equilibrium Equation 2 or Equation 4 to solve the entire model's endemic equilibrium. We note that for the stochastic model, this is not a stationary distribution, but a quasi-stationary distribution (QSD), as there exist absorbing states in the model.

To compute the QSD, note death (D) is an absorbing state and the set of transient states is $\mathcal{T} = (S, E_1, ..., E_n, I)$. Then the random variable representing time to absorption (death) as a phase-type distri-

bution. The QSD over $\mathcal{T}$ will arise by conditioning on survival. This distribution allows us to distribute mosquitoes across the transient states properly at equilibrium. We partition $\mathbf{Q}$ as [13, 14]:

$$\widetilde{\mathbf{Q}} = \begin{pmatrix} \mathbf{T} & t \\ \mathbf{0} & 0 \end{pmatrix} \tag{28}$$

Here, $\mathbf{T}_{|\mathcal{T}|\times|\mathcal{T}|}$ is a subintensity matrix of transition rates between transient states, and $t_{|\mathcal{T}|\times 1}$ is a column vector of exit rates to the absorbing state. We denote the random variable following a continuous phase-type distribution describing time until absorption as $\tau \sim \text{PH}(\pi, \mathbf{T})$ with density function $f_\tau(u) = \pi e^{\mathbf{T}u} t$, where $\pi_{1\times|\mathcal{T}|}$ is a row vector specifying the initial distribution over transient states.

Let $\mathbf{U} = (-\mathbf{T})^{-1}$ be the matrix containing the means of random variables u_{ij} denoting time spent in state j starting from i, prior to absorption. We can use this matrix to define the QSD over $\mathcal{T}$, denoted as $\tilde{\pi}$. The jth element of the quasi-stationary distribution is [15, 16]:

$$\widetilde{\pi}_j = \frac{\pi^\top \mathbf{U} f_j}{\pi^\top \mathbf{U} e} \tag{29}$$

In Equation 29, f_j is a column vector with 1 in the jth row and 0 elsewhere, and e is a column vector of 1's. In our specific case, π places all mass on state S because the mosquito cannot emerge from the pupa stage already infected (there is no vertical transmission of pathogen), so the quasi-stationary distribution can be directly obtained from $\mathbf{U}$.

2.3.1 Coupled SEI-SIS Equilibrium Solutions

In order to solve for the mosquito population required to produce some equilibrium prevalence in humans, let N_V be the total adult female population, summing over infection states. Writing the SIS human dynamics (Equation 21) with the expanded form of λ_H, we have:

$$\begin{aligned} \frac{d}{dt}S_H(t) &= \mu\big(S_H(t) + I_H(t)\big) - \mu S_H(t) - \left(abI_V\left(\frac{1}{S_H(t)+I_H(t)}\right)\right)S_H(t) + rI_H(t) \\ \frac{d}{dt}I_H(t) &= -\mu I_H(t) + \left(abI_V\left(\frac{1}{S_H(t)+I_H(t)}\right)\right)S_H(t) - rI_H(t) \end{aligned} \tag{30}$$

Because we consider both the human population size $N_H = S_H + I_H$ and equilibrium prevalence $x = \frac{I_H}{N_H}$ known constants, we can solve the model at equilibrium in terms of the number of infected mosquitoes, such that:

$$I_V = \frac{I_H(S_H + I_H)(r+\mu)}{abS_H} \tag{31}$$

After we have solved for the number of infected mosquitoes I_V, we can derive the total female mosquito population this implies:

$$
\begin{aligned}
I_V &= N_V \left(\frac{(n\gamma_V)^n \lambda_V}{(n\gamma_V + \mu_V)(\lambda_V + \mu_V)} \right) \\
N_V &= I_V \left(\frac{(n\gamma_V + \mu_V)(\lambda_V + \mu_V)}{(n\gamma_V)^n \lambda_V} \right)
\end{aligned} \tag{32}
$$

This total size N_V can be spread across the EIP stages via Equation 29. Given N_V, we can calculate equilibrium solutions for the full lifecycle model by plugging in this number of adult females into either Equation 2 or 4.

2.4 SEIR Model

We also present results for an SEIR style model of human dynamics. An additional parameter, γ_H, is the rate of progression from $E_H \to I_H$, that is, the inverse of the duration of latency in humans.

$$
\begin{aligned}
\frac{d}{dt} S_H &= \mu N_H - \lambda_H S_H - \mu S_H \\
\frac{d}{dt} E_H &= \lambda_H S_H - \gamma_H E_H - \mu E_H \\
\frac{d}{dt} I_H &= \gamma_H - r I_H - \mu I_H \\
\frac{d}{dt} R_H &= r I_H - \mu R_H
\end{aligned} \tag{33}
$$

Where N_H is the total human population and the force of infection on humans follows the Ross-Macdonald form $\lambda_H = abI_V \left(\frac{1}{N^H}\right)$. We consider that the total population size N_H and the number of infected and infectious humans I_H are known, allowing us to solve for R_H and E_H at equilibrium. It should be noted that for realistic parameter values, this model only has a non-trivial equilibrium when $\mu < \frac{\mu N_H - r I_H}{I_H}$, which results in extremely unrealistic (short) lifespans. The other two equilibrium points are the trivial disease free equilibrium when $\lambda_H = 0$ and the normal ($\lambda_H > 0$) case when $R_H(\infty) \to N_H$, that is, for realistic values of parameters, all surviving individuals will become infected subsequently recover. For that reason we do not explicitly calculate the endemic equilibrium. In general the SEIR human model should be used to evaluate the impact of gene drive interventions on one-off *epidemic* situations (eg; does releasing large numbers of modified mosquitoes 10 days after initial cases appear make a significant difference in final outcome), rather than for investigating *endemic* diseases, which require more complex models with waning immunity.

3 Stochastic Petri Net

Here we provide an introduction to the stochastic Petri net modeling formalism used in **MGDrivE 2**. Notation in this introduction is borrowed from [12].

3.1 Properties of SPN

SPN is a mathematical modeling language to describe discrete event systems, that is, a system which has a countable set of events (although only a finite number may enabled at any given time), each of which changes state in some way when it occurs. When events are assumed to happen after Exponentially-distributed

intervals (alternatively, each event occurs at a constant, age-independent rate), the SPN is isomorphic to a CTMC, and can be extended to provide a modeling semantics for generalized semi-Markov processes [17]. For practical application, a benefit of adopting the SPN modeling language is that model representation is separate from numerical simulation. This can allow both for highly efficient simulation, as the model can be represented via vectors and sparse matrices, and also utilization of model-agnostic simulation algorithms that take as input a generic SPN model and output sampled trajectories.

A Petri net is, formally, a bipartite graph, consisting of a set of *places*, $\mathcal{P}$, and a set of *transitions*, $\mathcal{T}$. Directed edges, often called *arcs*, lead from places to transitions and from transitions to places. Arcs are allowed to have a positive integer weight. Therefore, if $u = |\mathcal{P}|$ and $v = |\mathcal{T}|$, the set of arcs that connect places to transitions can be denoted by a non-negative integer matrix $\mathbf{Pre}_{v \times u}$, and the set of arcs connecting transitions to places by $\mathbf{Post}_{v \times u}$.

This bipartite graph so far defines the *structural* properties of the model. When translating conceptual models to the language of Petri nets, the places define the allowable state space of the model. However, in order to describe any particular state of the model, the Petri net must be given a *marking*, M, which is given by associating with each place a non-negative integer number of *tokens*, such that $M \in \mathbb{N}^u$. Put more concretely, we can imagine taking some number of indistinguishable tokens and assigning each one to a place in the set $\mathcal{P}$; the resulting vector in $\mathbb{N}^u$ is a valid state of the model. In the language of CTMCs the marking M is referred to as the *state*, and we use the terms interchangeably.

Each transition $k \in \mathcal{T}$ is allowed to change state when it occurs (fires). A transition k is *enabled* in some marking M and may fire when there are tokens on the place corresponding to each input arc (k^{th} row of $\mathbf{Pre}$) greater than or equal to the arc weight. When the transition fires, M is updated by removing tokens from M given by the set of input arcs and weights, that is, according to $\mathbf{Pre}$. It then adds tokens in M according to its output arcs and weights in $\mathbf{Post}$. This can be represented succinctly if we let $\mathbf{A} = \mathbf{Post} - \mathbf{Pre}$, then if r_k is a column vector of zeros with a one at place k, the state can be updated as $M' = M + \mathbf{S}r_k$, where $\mathbf{S} = \mathbf{A}^\intercal$. $\mathbf{S}$ has dimensions $u \times v$, so it maps vectors in the space of events to vectors in the space of marking updates.

So far we have described a deterministic Petri net. However, associate with each transition a clock that tells us when k will fire, if it were the first of all enabled transitions to fire and let the process associated with k be a Poisson process Y_k with intensity λ_k which may depend on the current time t, and current marking $M(t)$. Finally, if we let all enabled processes Y_k compete under a race condition by sampling the next firing time for each clock, τ_k, such that $k' = \arg\min_k\{\tau_k\}$, then k' is the event that fires. In that case the system time is updated to $t' = t + \tau_{k'}$ and the state as $M(t') = M(t) + \mathbf{S}r_{k'}$. It can be rigorously proven that such a construction is a continuous-time Markov chain [18].

An advantage of this construction of a Markov process rather than the more traditional presentation via the infinitesimal generator matrix is that processes with infinite state spaces can be compactly represented, because only a finite number of clock processes compete at any given time. In this way, infinite birth death processes, for example, can be succinctly represented graphically and simulated. Additionally, because most transitions only have a few input and output arcs, the matrices $\mathbf{Pre}$ and $\mathbf{Post}$, which define the bipartite graph, will be highly sparse.

3.2 SPN Architecture

We have developed algorithms to construct Petri nets for arbitrary genetic inheritance cubes [1], metapopulation structure, Erlang-distributed aquatic stages, infection dynamics, and human populations. Once built, and augmented with parameters for hazard functions in $\mathcal{T}$, the resulting SPN model can be numerically evaluated via a variety of sampling algorithms. We describe the SPN architecture without considering epidemiological dynamics, as those are considered in a later section.

MGDrivE 2 has been designed with consideration for computational efficiency. We store the matrices defining the SPN in sparse matrix format using the Matrix **R** package [19]. In addition, when constructing $\mathcal{T}$, we check the input inheritance cube. If the viability mask ($\overline{\overline{\overline{\Lambda}}}$) indicates a certain cross will never produce viable offspring, or if the probability of offspring for a cross is zero ($\overline{\overline{\overline{Ih}}}$), that transition is not instantiated in the SPN.

Generation of the set of places $\mathcal{P}$ for a single node is simple, and requires the user to pass the inheritance cube $\overline{\overline{\overline{Ih}}}$, and the shape parameters for Erlang dwell distributions associated with egg, larval, and pupal aquatic stages to the function. The function returns the named set of places, along with an indexing data structure containing the indices of places stratified by life stage and genotype, both for easy debugging and the construction of arcs when the set of transitions is made. We note that SPN defines no particular order on the set $\mathcal{P}$ but we "unroll" the set hierarchically into a vector first by node, life-stage, and genotype, for easier comprehension and testing.

After $\mathcal{P}$ is constructed, we can construct transitions $\mathcal{T}$, using $\mathcal{P}$ as input, as well as the aforementioned shape parameters of aquatic dwell times and $\overline{\overline{\overline{Ih}}}$. While not strictly necessary for SPN, we adopted several conventions here which simplify later generation of hazard functions. We first defined "classes", K, of transitions, such that, for example, all transitions related to oviposition were grouped together. Each class K then is a proper subset of all transitions, $K \subset \mathcal{T}$, and $\bigcup_i K_i = \mathcal{T}$. Each individual transition in a set $k_j \in K_i$ has an associated **R** function which returns a data structure containing, at minimum, an index `vix`, indicating where in $\mathcal{T}$, k_j can be found, a `label`, a character string giving the name of the transition, `s` and `s_w`, indices of input arcs (the places they originate at), and weights respectively, `o` and `o_w`, the same for output arcs from this transition back to places, and `class`, giving the name of the class K_i as a character string this transition belongs to. To make $\mathcal{T}$, we iterate through classes K_i, and k_j within classes, storing each transition's packet of information in the vector $\mathcal{T}$. Because each transition "knows" its input and output arcs, as well as their weights, adding new classes of transitions is simple, as a single function merely needs to be written that takes in places and perhaps genetic information, and returns this minimal packet of information.

Once $\mathcal{P}$ and $\mathcal{T}$ are constructed, the SPN is formally constructed. However for computation, we prefer to store a more compact representation of the net. It is at this point we build the sparse matrices **Pre** and **Post**. To do so, we simply allocate two $v \times u$ sparse integer matrices, then iterate through $k \in \mathcal{T}$. We use the information packet described earlier, specifically `s`, `s_w` and `o`, `o_w`, to fill in the non-zero entries of **Pre** and **Post** respectively. Because we have already induced ordering on $\mathcal{P}$ and $\mathcal{T}$, the matrices have the right sorting of rows and columns.

3.3 Hazard Functions

To build a CTMC model from the Petri net (SPN), each transition must have a hazard function $\lambda_k(t, M(t))$. In the code implementation of **MGDrivE 2**, each hazard first checks if that transition is enabled, if not it immediately returns zero and else computes the hazard rate. We allow λ_k to be a function of time for simulation of inhomogeneous processes. Because in this manuscript we consider only Markovian systems, hazards only depend on the current system state $M(t)$ and time t. Unless otherwise noted, we use λ_k to generically denote the joint hazard and enabling function for process Y_k.

This use of CTMC is known as a Markov population process, and has been used to model stochastic population models for some time now [20]. However, it can be useful to include exogeneous stochastic processes into the model, which may affect hazard rates. These processes could represent, for example, environmental processes such as temperature or rainfall. Knowledge of these processes would be necessary to evaluate the hazard functions. Consider the situation in which process Y_k is affected by environmental stochasticity represented by z so that the hazard is $\lambda_k(t, M(t), z)$; for concreteness, consider z to be temperature and k to be larval mortality. We must also consider a specific function of interest (f) to be computed from trajectories

of **MGDrivE 2**, which we would like to estimate via Monte Carlo, to average over uncertainty in z. Again for concreteness, we could consider functions like time required for a specific gene to fixate, or time required for pathogen extinction in a specific node. To propagate uncertainty from arbitrary exogenous processes, we simply draw many samples from z and then run Monte Carlo simulation of **MGDrivE 2** on each realized exogenous trajectory; we can imagine an "outer" loop sampling a trajectory $\tilde{z}$ from z and an "inner" loop computing Monte Carlo estimates of f, conditioning on $\tilde{z}$ as deterministic input to $\lambda_k(t, M(t), \tilde{z})$. Because z is by definition an exogeneous source of stochastic variation, the probability factorizes such that this method properly propagates uncertainty into our estimation of f. For specific functions, more efficient methods than naive Monte Carlo may exist, and we refer to the panoply of variance-reduction methods covered in [21].

In **MGDrivE 2**, once the Petri net $(\mathcal{P}, \mathcal{T})$ is constructed (and we have parameters θ and inheritance cube $\overline{\overline{\overline{Ih}}}$), we can construct the v-dimensional column vector of hazard functions Λ. Specifically, we store the individual λ_k functions as function closures within the vector Λ, as functions are first class objects in **R**. We note that this can be easily adapted to other programming languages, for example in C++98, functors could be used in lieu of closures, and in C++11/14, lambda functions could achieve the same effect [22]. Each closure stores only the elements of θ and $\overline{\overline{\overline{Ih}}}$ necessary for computation of the hazard. We note that the function closure based storage of hazard functions means that it is easy to include additional computational state for specialized algorithms or more complex processes, such as enabling times or integrated hazards.

So far we have described what the fully constructed object Λ is, but not yet how we implemented it in code, which we do now. Much like the construction of $\mathcal{T}$, we rely heavily on our assignment of transitions into classes; after allocating memory for a v-length vector, we begin iterating through $k \in \mathcal{T}$. First, we scan the `class` of the transition; this tells us the appropriate function factory to call that will return λ_k, the function closure that computes the hazard. We pass the packet k to the function factory, along with parameters θ. The packet provides all the necessary information to set up the enabling rule, and the function factory pulls out the necessary components of θ to compute that specific hazard, which are stored in the function closure. All returned hazard functions λ_k accept only two arguments, t time, and M, the state (marking).

We allow the option for users to select if the vector of hazard functions shall be "exact" or "approximate". Exact hazards are required for both sampling algorithms that simulate integer numbers of tokens, in which case enabling rules make sense. In this case evaluation of λ_k proceeds as described above. If however a continuous-state approximation is desired, either from a deterministic interpretation of the hazard functions as rate functions for mean-field approximation, or from a drift-diffusion stochastic differential equation, we ignore the check for sufficient tokens on input arcs, as the model no longer has an integer state space.

3.4 Numerical Simulation

One key feature of the SPN representation of **MGDrivE 2** is the convenient decoupling of model specification and sampling method, allowing model-independent development of fast algorithms. This lets us benefit from extensive work into optimized stochastic sampling algorithms from the chemical kinetics and physical simulation communities, many of which can be used nearly "off the shelf" with a place/transition model representation. We refer to the encyclopedic book by [23] as one of many resources for fast simulation routines.

Currently we do not support exact simulation of inhomogeneous processes, although approximate simulation is best done via the Poisson time-step method, where inhomogeneous terms are discretized to a piecewise constant step function with the same Δt as used in the approximate time-step. Exact simulation of inhomogeneous processes is difficult, although an algorithm based on random time change was presented by [24], and [25] investigate exact and approximate rejection-based methods. We leave the incorporation of these or similar sampling methods into the **MGDrivE 2** framework for future development.

We provide example code to numerically integrate deterministic trajectories, based on the deSolve **R**

package of ODE solvers [26], using a mean-field approximation to the stochastic system [27]. We also provide several stochastic samplers for both exact and approximate trajectories, inspired by the smfsb **R** package [12].

In certain situations, when populations are large (that is, no places have a small number of tokens) and hazard functions are close to linear (guaranteed when using mass-action forms), it may be the case that stochastic fluctuations can be safely neglected. [3] made rigorous the conditions under which CTMCs may converge to ODEs. Such an approach essentially simplifies to considering the hazard functions as rate functions, and the state $M(t)$ as a continuous quantity (motivating the ability for the user to select generation of "approximate" Λ) [28]. Even when stochastic fluctuations are non-negligible such that deterministic approximation is not valid, it may be useful to provide the option for deterministic integration of the SPN model for quick visualization of transient behavior, or for sensitivity analysis.

At present we only offer Gillespie's "direct-method" to sample statistically exact trajectories, a well known method to sample from stochastic models [29]. Being an exact sampler, it samples integer-valued trajectories, and thus uses exact hazards and enabling functions. Briefly, the method works via a simple update step where first the vector of hazard functions is evaluated, $h(t) = \Lambda(t, M(t))$. The Markov transition kernel to the next state can be factored such that the sampler first samples the random variable τ describing *when* the jump occurs, relative to t, $\tau \sim \text{Exp}\left(\sum_k h_k(t)\right)$. Next it samples *which* process caused the jump, and updates the system accordingly, that is, it selects the process causing the jump, k' with probability $\frac{h_{k'}(t+\tau)}{\sum_k h_k(t+\tau)}$. Then update the marking according to matrix equation $M'(t+\tau) = M(t) + \mathbf{S}r_{k'}$. In general, large populations of mosquitoes and/or large numbers of nodes would render exact simulation practically impossible, if, for example many tens of thousands of individual events needed to fire each day, each requiring a system update and resampling of random variables for each event, both of which are computationally expensive tasks.

In addition, there are two approximate stochastic sampling algorithms that have been implemented for use in **MGDrivE 2**, and we anticipate future algorithmic development focusing on implementation of improved approximate samplers. The first of these is a simple fixed size tau-leaping method, the Poisson time-step (PTS), reviewed in [12] and first introduced by [30]. The basic concept behind the PTS algorithm is that if none of the hazard functions change significantly over a small time step, say $[t, t + \Delta t)$, then one can approximate the state change by sampling a Poisson distributed random variable for enabled each $k \in \mathcal{T}$, such that the elements of the r vector indicating how many times each event fired are each independent Poisson random variates with rate parameter $\lambda_k(t, M(t))\,\Delta t$. Then the matrix update can be preformed with those sampled Poisson variates in the vector r, system time updated, and another iteration preformed. The extent to which the assumption that hazards do not change significantly over the interval determines the quality of the approximation. The original tau-leaping algorithm has spawned many variations on the theme, including some with strong probabilistic guarantees of approximation quality [31], which may be incorporated for **MGDrivE 2**.

The second approximate stochastic sampling algorithm is based on a continuous state stochastic differential equation (SDE), known as the diffusion approximation. We offer a brief heuristic explanation of the algorithm but a detailed derivation can be found in [32]. If one starts with the integer valued Markov jump process, it will have a set of differential equations known as Kolmogorov's forward equations (KFE), sometimes referred to as (chemical) Master equation. The KFE gives the complete time-evolution of the probability mass function across allowable system states, and is usually intractable. It is possible (see [33] for a brief overview) to generate a partial differential equation (PDE) second-order approximation to the KFE known as the Fokker-Planck equation, which approximates the probability mass function with a probability density function evolving according to first order drift and second order diffusion coefficients. Considering the marking M as a continuous state, the Fokker-Planck equation can be interpreted as a u-dimensional SDE driven by independent Wiener processes. While advanced techniques for simulation of SDEs exist [34], we implement the simple Euler-Maruyama method in **MGDrivE 2**.

References

[1] Héctor M. Sánchez C. et al. "MGDrivE: A modular simulation framework for the spread of gene drives through spatially explicit mosquito populations". In: *Methods in Ecology and Evolution* (2019). ISSN: 2041210X. DOI: 10.1111/2041-210X.13318.

[2] Paul J Hurtado and Adam S Kirosingh. "Generalizations of the 'Linear Chain Trick': incorporating more flexible dwell time distributions into mean field ODE models". In: *Journal of mathematical biology* 79.5 (2019), pp. 1831–1883.

[3] Thomas G. Kurtz. "Solutions of Ordinary Differential Equations as Limits of Pure Jump Markov Processes". In: *Journal of Applied Probability* 7.1 (1970), p. 49. ISSN: 00219002. DOI: 10.2307/3212147. URL: http://www.jstor.org/stable/3212147?origin=crossref.

[4] Penny A Hancock and H Charles J Godfray. "Application of the lumped age-class technique to studying the dynamics of malaria-mosquito-human interactions". In: *Malaria journal* 6.1 (2007), p. 98.

[5] John M Marshall, Anna Buchman, Omar S Akbari, et al. "Overcoming evolved resistance to population-suppressing homing-based gene drives". In: *Scientific reports* 7.1 (2017), p. 3776.

[6] Peter G Fennell, Sergey Melnik, and James P Gleeson. "Limitations of discrete-time approaches to continuous-time contagion dynamics". In: *Physical Review E* 94.5 (2016), p. 052125.

[7] Linda JS Allen. "Some discrete-time SI, SIR, and SIS epidemic models". In: *Mathematical biosciences* 124.1 (1994), pp. 83–105.

[8] Anne Deredec, H Charles J Godfray, and Austin Burt. "Requirements for effective malaria control with homing endonuclease genes". In: *Proceedings of the National Academy of Sciences* 108.43 (2011), E874–E880.

[9] Nikolay P Kandul et al. "Transforming insect population control with precision guided sterile males with demonstration in flies". In: *Nature communications* 10.1 (2019), p. 84.

[10] Maia Martcheva. *An introduction to mathematical epidemiology.* Vol. 61. Springer, 2015.

[11] David L Smith and F Ellis McKenzie. "Statics and dynamics of malaria infection in Anopheles mosquitoes". In: *Malaria journal* 3.1 (2004), p. 13.

[12] Darren James Wilkinson. *Stochastic Modelling for Systems Biology.* 2006. ISBN: 1-58488-540-8. DOI: 10.1080/09332480.2012.752295.

[13] Mogens Bladt and Bo Friis Nielsen. *Matrix-Exponential Distributions in Applied Probability.* Vol. 81. 2017. ISBN: 978-1-4939-7047-6. DOI: 10.1007/978-1-4939-7049-0. URL: http://link.springer.com/10.1007/978-1-4939-7049-0.

[14] Peter Buchholz, Jan Kriege, and Iryna Felko. *Input Modeling with Phase-Type Distributions and Markov Models.* 2014. ISBN: 978-3-319-06673-8. DOI: 10.1007/978-3-319-06674-5. URL: http://link.springer.com/10.1007/978-3-319-06674-5.

[15] J. N. Darroch and E. Seneta. "On Quasi-Stationary Distributions in Absorbing Discrete-Time Finite Markov Chains". In: *Journal of Applied Probability* 4.1 (1965), p. 192. ISSN: 00219002. DOI: 10.2307/3212311. URL: http://www.jstor.org/stable/3212311?origin=crossref.

[16] J. N. Darroch and E. Seneta. "On Quasi-Stationary Distributions in Absorbing Continuous-Time Finite Markov Chains". In: *Journal of Applied Probability* (1967). ISSN: 00219002. DOI: 10.2307/3212311.

[17] Peter W. Glynn. "A GSMP Formalism for Discrete Event Systems". In: *Proceedings of the IEEE* (1989). ISSN: 15582256. DOI: 10.1109/5.21067.

[18] Pierre Brémaud. *Markov chains : Gibbs fields, Monte Carlo simulation, and queues.* 1999. ISBN: 0387985093 (acid-free paper).

[19] Douglas Bates and Martin Maechler. *Matrix: Sparse and Dense Matrix Classes and Methods.* R package version 1.2-17. 2019. URL: https://CRAN.R-project.org/package=Matrix.

[20] John F.C. Kingman. "Markov Population Processes". In: *J. Appl. Probab.* 6.1 (1969), pp. 1–18. ISSN: 0021-9002. URL: http://www.jstor.org/stable/3212273.

[21] Paul Bratley, Bennet Fox, and Linus Schrage. *A guide to simulation.* Springer Science & Business Media, 2011.

[22] Scott Meyers. *Effective modern C++: 42 specific ways to improve your use of C++ 11 and C++ 14.* O'Reilly Media, Inc., 2014.

[23] Luca Marchetti, Corrado Priami, and Vo Hong Thanh. *Simulation algorithms for computational systems biology.* Springer, 2017.

[24] David F. Anderson. "A modified next reaction method for simulating chemical systems with time dependent propensities and delays". In: *Journal of Chemical Physics* 127.21 (2007). ISSN: 00219606. DOI: 10.1063/1.2799998. arXiv: 0708.0370.

[25] Vo Hong Thanh et al. "Incorporating extrinsic noise into the stochastic simulation of biochemical reactions: A comparison of approaches". In: *Journal of Chemical Physics* 148.6 (2018). ISSN: 00219606. DOI: 10.1063/1.5016338. URL: http://dx.doi.org/10.1063/1.5016338.

[26] Karline Soetaert, Thomas Petzoldt, and R. Woodrow Setzer. "Solving Differential Equations in R : Package deSolve". In: *Journal of Statistical Software* (2010). ISSN: 1548-7660. DOI: 10.18637/jss.v033.i09.

[27] Luca Bortolussi et al. "Continuous approximation of collective system behaviour: A tutorial". In: *Performance Evaluation* 70.5 (May 2013), pp. 317–349. ISSN: 01665316. DOI: 10.1016/j.peva.2013.01.001. URL: http://linkinghub.elsevier.com/retrieve/pii/S0166531613000023.

[28] Daniel T. Gillespie. "Stochastic simulation of chemical kinetics". In: *Ann. Rev. OF Phys. Chem.* 58.1 (May 2007), pp. 35–55. ISSN: 0066-426X. DOI: 10.1146/annurev.physchem.58.032806.104637. URL: http://www.annualreviews.org/doi/10.1146/annurev.physchem.58.032806.104637.

[29] Daniel T. Gillespie. "Exact stochastic simulation of coupled chemical reactions". In: *The journal of physical chemistry* 81.25 (1977), pp. 2340–2361.

[30] Daniel T. Gillespie. "Approximate accelerated stochastic simulation of chemically reacting systems". In: *The Journal of Chemical Physics* 115.4 (2001), pp. 1716–1733.

[31] David F. Anderson. "Incorporating postleap checks in tau-leaping". In: *Journal of Chemical Physics* 128.5 (2008). ISSN: 00219606. DOI: 10.1063/1.2819665.

[32] Daniel T. Gillespie. "The multivariate Langevin and Fokker–Planck equations". In: *American Journal of Physics* 64.10 (2005), pp. 1246–1257. ISSN: 0002-9505. DOI: 10.1119/1.18387.

[33] Raúl Toral and Pere Colet. *Stochastic numerical methods: an introduction for students and scientists.* John Wiley & Sons, 2014.

[34] Simo Särkkä and Arno Solin. *Applied stochastic differential equations.* Vol. 10. Cambridge University Press, 2019.
